# Role of Long Non-Coding RNA X-Inactive-Specific Transcript (*XIST*) in Neuroinflammation and Myelination: Insights from Cerebral Organoids and Implications for Multiple Sclerosis

**DOI:** 10.3390/ncrna11030031

**Published:** 2025-04-29

**Authors:** Nihan Aktas Pepe, Busra Acar, Gozde Erturk Zararsiz, Serife Ayaz Guner, Alaattin Sen

**Affiliations:** 1 Department of Molecular Biology and Genetics, Faculty of Life and Natural Sciences, Abdullah Gul University, Kayseri 38080, Türkiye; nihan.aktas@agu.edu.tr (N.A.P.); busra.acar@agu.edu.tr (B.A.); 2 Department of Biostatistics, Faculty of Medicine, Erciyes University, Kayseri 38039, Türkiye; gozdeerturk@erciyes.edu.tr; 3 Department of Molecular Biology and Genetics, Faculty Sciences, Izmir Institute of Technology, Izmir 35430, Türkiye; serifeayaz@iyte.edu.tr; 4 Department of Biology, Faculty of Sciences, Pamukkale University, Kinikli, Denizli 20070, Türkiye

**Keywords:** *XIST*, long non-coding RNA, cerebral organoids, neuroinflammation, myelination

## Abstract

**Background/Objectives**: X-inactive-specific transcript (*XIST*) is a factor that plays a role in neuroinflammation. This study investigated the role of *XIST* in neuronal development, neuroinflammation, myelination, and therapeutic responses within cerebral organoids in the context of Multiple Sclerosis (MS) pathogenesis. **Methods**: Human cerebral organoids with oligodendrocytes were produced from *XIST*-silenced H9 cells, and the mature organoids were subsequently treated with either FTY720 or DMF. Gene expression related to inflammation and myelination was subsequently analyzed via qRT-PCR. Immunofluorescence staining was used to assess the expression of proteins related to inflammation, myelination, and neuronal differentiation. Alpha-synuclein protein levels were also checked via ELISA. Finally, transcriptome analysis was conducted on the organoid samples. **Results**: *XIST*-silenced organoids presented a 2-fold increase in the expression of neuronal stem cells, excitatory neurons, microglia, and mature oligodendrocyte markers. In addition, *XIST* silencing increased IL-10 mRNA expression by 2-fold and MBP and PLP1 expression by 2.3- and 0.6-fold, respectively. Although *XIST* silencing tripled IBA1 protein expression, it did not affect organoid MBP expression. FTY720, but not DMF, distinguished MBP and IBA1 expression in *XIST*-silenced organoids. Furthermore, *XIST* silencing reduced the concentration of alpha-synuclein from 300 to 100 pg/mL, confirming its anti-inflammatory role. Transcriptomic and gene enrichment analyses revealed that the differentially expressed genes are involved in neural development and immune processes, suggesting the role of *XIST* in neuroinflammation. The silencing of XIST modified the expression of genes associated with inflammation, myelination, and neuronal growth in cerebral organoids, indicating a potential involvement in the pathogenesis of MS. **Conclusions**: *XIST* may contribute to the MS pathogenesis as well as neuroinflammatory diseases such as and Alzheimer’s and Parkinson’s diseases and may be a promising therapeutic target.

## 1. Introduction

X-inactive-specific transcript (*XIST*) is a collection of 15,000–20,000 nucleotide sequences found in the q13.2 X-inactivation center [1,2]. Previous research has demonstrated that *XIST* controls X chromosome inactivation (XCI) and contributes to the heritable silencing of one of the X chromosomes during the formation of female cells [3]. The literature provides a thorough understanding of the role of *XIST* in multiple cellular functions, such as apoptosis, differentiation, proliferation, adhesion, migration, inflammation, and polarization. This influence is exerted through the modulation of multiple signaling pathways beyond its XCI role [4,5]. In addition, *XIST* plays a role in the development of intricate disorders by acting as a competitive endogenous RNA (ceRNA). Furthermore, *XIST* has been linked to complex disorders such as Alzheimer’s disease (AD), neurodegeneration, and neuroinflammation [6,7,8,9]. An increasing body of evidence suggests that *XIST* may play a role in the onset and progression of autoimmune and neurodegenerative diseases, either as a regulator of miRNA-mRNA interactions or through other mechanisms [5,10].

Chronic neuroinflammatory and neurodegenerative disorders affect more than 50 million individuals globally [11]. Neuroinflammatory disorders may disproportionately impact women [11,12]. This sex bias might stem from genetic and environmental factors [13,14]. Despite the expectation that genes localized on the X chromosome would be silenced with *XIST*, 11–30% of human X-related genes manage to evade XCI and remain somewhat expressed. This phenomenon is called XCI escape [15]. Previous research has revealed that several XCI-escaping genes, such as *CD40L, IRAK-1, KDM6A*, and *TLR7*, are linked to autoimmunity and neuroinflammation [16,17]. Recent advances in our comprehension of lncRNA function have revealed that dysfunction of *XIST* may be closely linked to the pathogenesis of several neurodegenerative and autoimmune diseases. In particular, aberrant *XIST* expression or localization has been implicated in conditions such as AD, Parkinson’s disease (PD), and Multiple Sclerosis (MS), where dysregulation of *XIST* may contribute to altered immune responses and neuronal degeneration through mechanisms including the modulation of miRNA interactions and chromatin remodeling [7,18,19,20,21].

Recent studies underscore that *XIST* is not merely a housekeeping epigenetic molecule but one that intersects with pathways of immune signaling, microglial polarization, and cytokine production [22,23,24]. In the context of MS, *XIST*’s expression and function appear to be dysregulated, contributing to the disease’s immunopathology—for example, through the *XIST*–miR-326–HNRNPA1 axis that may centrally drive MS-related gene networks [25]. Disease-modifying therapies (DMTs) are used to treat MS to reduce relapses and slow disease progression by regulating inflammatory and immune responses in MS patients rather than providing a total cure for the disease [26]. Among these DMTs, fingolimod (FTY720, Gilenya) is a synthetic analogue of sphingosine and was the first to be licensed by the Food and Drug Administration for the oral treatment of MS. Once phosphorylated, it binds to sphingosine-1-phosphate receptors (S1PRs) and causes the internalization and degradation of S1PR1. Hence, T and B cells in the blood and those that invade the CNS are both decreased by FTY720 [27,28]. Dimethyl fumarate (DMF) is another oral DMT used for MS. Although the exact mechanism of action of DMF is yet unknown, it has been demonstrated that it reduces the activation of T cells and changes the distribution of helper T cells in addition to activating antioxidative and anti-inflammatory genes [29,30,31,32,33].

This study aimed to use cerebral organoids to examine the impact of the long non-coding RNA *XIST* on the development of neuroinflammation and myelination in the context of MS pathogenesis. First, *XIST*-siRNA was used to silence *XIST* expression in H9 human embryonic stem cells (hESCs). Subsequently, myelinated cerebral organoids were generated from *XIST*-silenced and control (scramble) H9 hESCs. To model neuroinflammation, LPS was applied to myelinated cerebral organoids. To determine the differential expression of inflammation- and myelination-related genes in the scramble- and *XIST*-silenced organoids, qRT-PCR, transcriptome, and immunofluorescence analyses were performed. For further elucidation, scramble- and *XIST*-silenced organoids were treated with either FTY720 or DMF to investigate their response to DMTs, and the expression levels of inflammation- and myelination-related genes were determined at both the mRNA and protein levels via qRT-PCR and immunofluorescence staining, respectively. While many previous studies have relied on traditional cell culture methods, the use of human cerebral organoids represents a significant advancement in modeling the complex three-dimensional architecture and cell–cell interactions of the human brain. These organoids provide a more physiologically relevant system to investigate the molecular underpinnings of *XIST* dysfunction. However, we acknowledge that further validation in animal models is essential to fully translate these findings to in vivo context. By integrating data from both advanced organoid models and animal studies, future research can more comprehensively elucidate the role of *XIST* in neuroinflammation and neurodegeneration.

## 2. Results

We have taken numerous steps to eliminate possible sources of bias as well as carefully verify our experimental assumptions so that our analysis would produce consistent causal inferences. Our study, for instance, used various independent controls—specifically MOCK-transfected and scramble-transfected H9 cells—to reduce confounding influences; all experiments were conducted in at least duplicate with suitable statistical analyses (e.g., two-way ANOVA and Student’s *t*-test) to guarantee reproducibility and reliability (see Section 2.1 and Section 2.2). The strength of our results is also confirmed by the morphological and molecular validation of cerebral organoid creation shown by immunofluorescence labeling and qRT-PCR studies. lncRNAs are defined as transcripts longer than 200 nucleotides that are not protein-coding but rather vital for the control of gene expression at the epigenetic, transcriptional, and post-transcriptional levels. As detailed in our Introduction (Pages 1–3, References [1,2,3,4,5]), a paradigmatic lncRNA *XIST* is mostly recognized for its involvement in X chromosomal inactivation—mediating gene silence by the recruitment of chromatin modifying complexes. We guarantee that our findings and causal readings on the function of *XIST* in myelination and neuroinflammation are both well founded and replicable by clarifying these definitions and outlining our strategies to reduce biases.

### 2.1. Myelinated and LPS-Induced Human Cerebral Organoids Were Generated from H9 Cells Transfected with XIST-siRNA or Scramble

**Figure 1:**
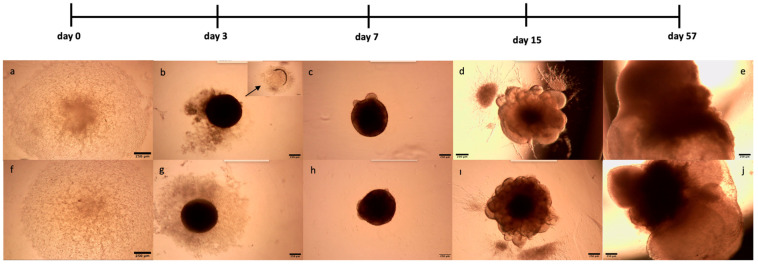
Generation of myelinated and LPS-induced human cerebral organoids from *XIST*-siRNA- and scramble-transfected H9 cells. *XIST*-siRNA (**a**)- and scramble (**f**)-transfected H9 cells were seeded into a U-bottom 96-well plate (day 0). (**b**,**g**). Observation of EBs obtained from *XIST*-siRNA (**b**)- and scramble (**g**)-transfected H9 cells in U-bottom plates. The arrow shows the empty area after the EBs were embedded in Matrigel (**c**,**h**). EBs obtained from the *XIST*-siRNA (**c**) and scramble (**h**) groups were embedded in Matrigel, and radial organization was observed in the EBs 3 days after incubation with neural induction medium (**d**,**i**). Culturing of EBs obtained from *XIST*-siRNA (**d**) and scramble (**i**) transfected in neural differentiation medium with vitamin A supplemented with growth factors and observation of small buds in EBs (**e**,**j**). Observation of myelinated and LPS-induced human cerebral organoids obtained from *XIST*-siRNA (**e**)- and scramble (**j**)-transfected control H9 cells. The images were taken with a Zeiss PrimoVert microscope with a Dino Capture USB microscope camera at 4× magnification. Scale bars indicate 250 µm.

Subsequent to transfection, the expression of *XIST* and the *XIST*-related genes FOXP3 and NANOG was compared via qRT-PCR to confirm the success of transfection prior to organoid production. Compared with MOCK, *XIST*-siRNA transfection reduced *XIST* expression 20-fold (Figure S1). The experimental setup used MOCK-transfected, *XIST*-siRNA-transfected, and scramble-transfected H9 cells to generate organoids. The transfection reagent was tested in MOCK-treated H9 hESCs (Figure S2). Since the MOCK-treated and control groups were similar, only the *XIST*-transfected and scramble groups were used for further research. Transfection with *XIST*-siRNA decreased *XIST* expression and increased FOXP3 expression in H9 cells, indicating successful *XIST* silencing in H9 hESC cells prior to organoid generation. The formation of cerebral organoids from both MOCK- and *XIST*-siRNA-transfected hESCs is shown in Figure 1. Both *XIST*-siRNA-transfected and scrambled H9 hESC tissues presented morphological changes consistent with Lancaster and Knoblich’s developmental processes and an optimized protocol [34,35]. These findings confirmed that organoid generation was successful.

### 2.2. Silencing of XIST Increased the Abundance of Neural Stem Cells, Excitatory Neurons, Mature Oligodendrocytes, and Microglia

**Figure 2:**
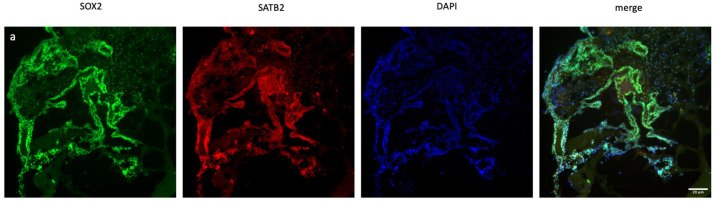

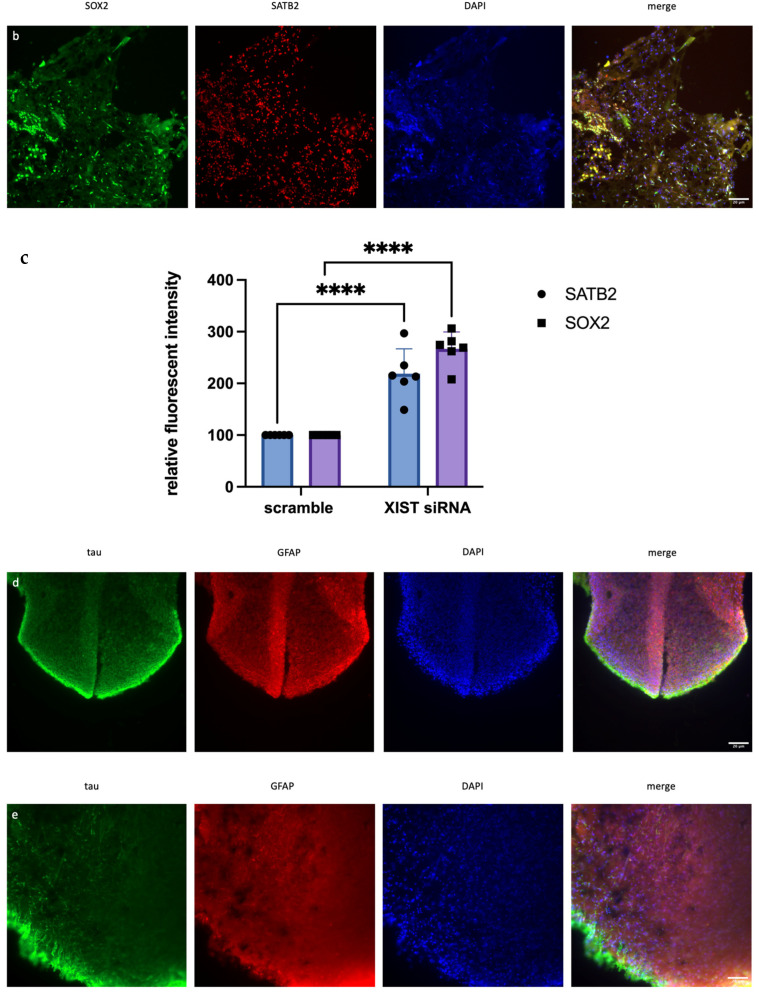

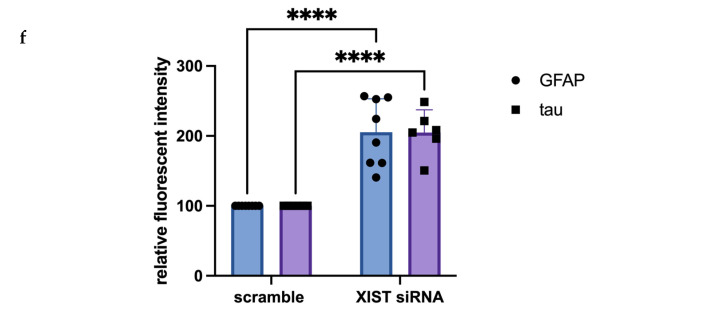
Immunofluorescence staining of scramble- and *XIST*-silenced organoid sections. (**a**,**b**) Immunofluorescence staining of organoid sections obtained from scramble- and *XIST*-siRNA-transfected H9 cells. Blue: DAPI (nucleus), red: SATB2 (excitatory neuron), and green: SOX2 (neural stem cell). Images labeled (**a**) are broad sections, whereas images labeled (**b**) are magnified views of a specific area from the general section. (**d**,**e**) Immunofluorescence staining of organoid sections obtained from scramble- and *XIST*-siRNA-transfected H9 cells. Blue: DAPI (nucleus), red: GFAP (astrocyte), and green: tau (mature oligodendrocyte). Relative fluorescence intensity of SOX2-, SATB2-, tau-, and GFAP-specific antibodies. Images labeled (**d**) are broad sections, whereas images labeled (**e**) are magnified views of a specific area from the general section. The results were calculated relative to those of the scramble group and then normalized to those of the DAPI group. Relative fluorescence intensity was calculated in tissue sections obtained from two independent experiments, and three technical replicates of the experiment for each sample were performed for each staining (**c**,**f**). The results obtained from two independent experiments were imported into GraphPad, and two-way ANOVA was performed on these two independent experiments (ns = *p* > 0.05, * = *p* ≤ 0.05, ** = *p* ≤ 0.01, *** = *p* ≤ 0.001, and **** = *p* ≤ 0.0001). The images were taken with a Leica DM6 B microscope at 20× and 40× magnification. Scale bars indicate 20 µm.

Immunofluorescence staining was used to assess human cerebral organoid formation, as well as the localization and expression of neuronal markers. First, the neural stem cell marker SOX2 and the excitatory and late-born superficial layer marker SATB2 are localized [36,37]. SOX2+ cells were mostly detected inside the organoid structure, whereas SATB2+ excitatory neurons were mostly localized on the outer surface (Figure 2a,b and Table S6). To conduct a more thorough examination of neuronal activity and the impact of *XIST* silencing, the localization and quantity of astrocytes and mature oligodendrocytes were assessed via GFAP and TAU immunostaining, respectively (Figure 2c,d). Figure 2e shows the calculated relative fluorescence intensity, indicating that cerebral organoids in which the *XIST* gene was silenced presented two-fold higher levels of GFAP, an astrocyte protein, and tau, a mature oligodendrocyte protein.

### 2.3. Silencing of XIST Alters the Expression Levels of Myelination- and Inflammation-Related Genes and the Response to DMTs

**Figure 3:**
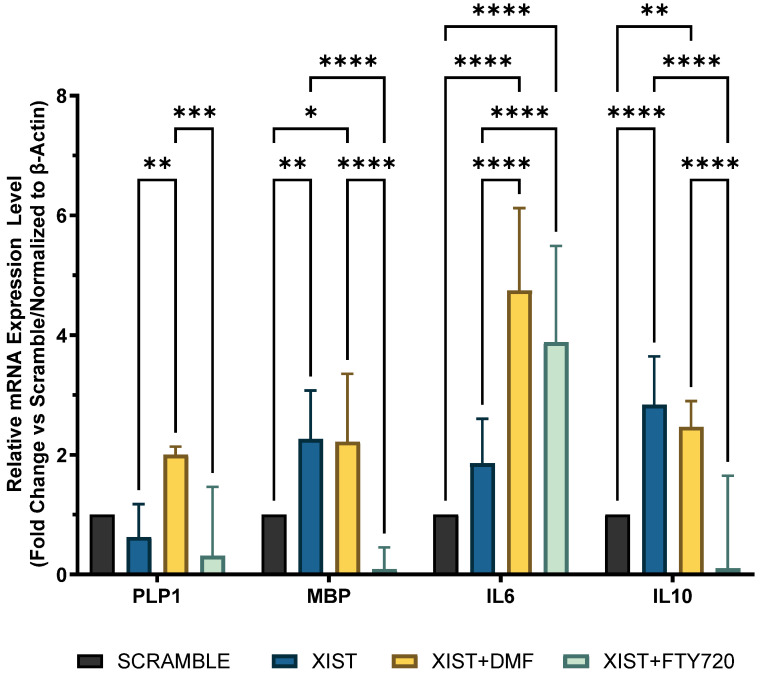
Determination of immune and myelination-related gene expression in scramble- and *XIST*-silenced organoids. Fold change in the expression levels of myelination-related *MBP* and *PLP1* genes and inflammation-related *IL10* and *IL6* genes in *XIST*-siRNA-transfected organoids compared with those in scramble-transfected organoids with or without treatment with either DMF or FTY720. Gene expression was normalized to that of the ß-actin housekeeping gene. Four replicates of the experiment (*n* = 4), each with duplicate measurements, were performed for each sample, and the fold-change of each gene of interest was calculated via Qiagen, Genelglobe. The results obtained from four independent experiments were imported into GraphPad, and two-way ANOVA test was performed on these four independent experiments (*n* = 4) (ns = *p* > 0.05, * = *p* ≤ 0.05, ** = *p* ≤ 0.01, *** = *p* ≤ 0.001, and **** = *p* ≤ 0.0001).

Since the inflammatory response and myelination regulate neurodegenerative pathogenesis of MS, the differential expression of selected immune- and myelin-related genes was determined in scramble- and *XIST*-silenced organoids. In *XIST*-siRNA-transfected organoids, myelin basic protein (*MBP*) expression was increased 2.26-fold; however, it did not significantly affect proteolipid protein 1 (*PLP1*) expression compared with that of *MBP* (Figure 3). *XIST* silencing altered *IL-10* and *IL-6* mRNA expression in cerebral organoids, affecting inflammatory responses. *IL-6* and *IL-10* expression was greater in organoids derived from *XIST*-silenced H9 hESCs than in those derived from scramble H9 cells (Figure 3). To determine how *XIST* silencing affects the DMT response, scramble and *XIST*-silenced organoids were treated with DMF or FTY720. DMF increased *MBP* and *PLP1* mRNA expression compared with that in the scramble group, whereas FTY720 decreased their levels after *XIST* silencing (Figure 3). *XIST* silencing significantly increased both pro- and anti-inflammatory cytokines (*IL-6* and *IL-10*) after DMF treatment. The mRNA expression of *IL-10* and *IL-6* exhibited opposing responses to FTY720 following *XIST* silencing (Figure 3).

### 2.4. Silencing of XIST Activates Microglia Without Altering MBP Protein Expression

**Figure 4:**
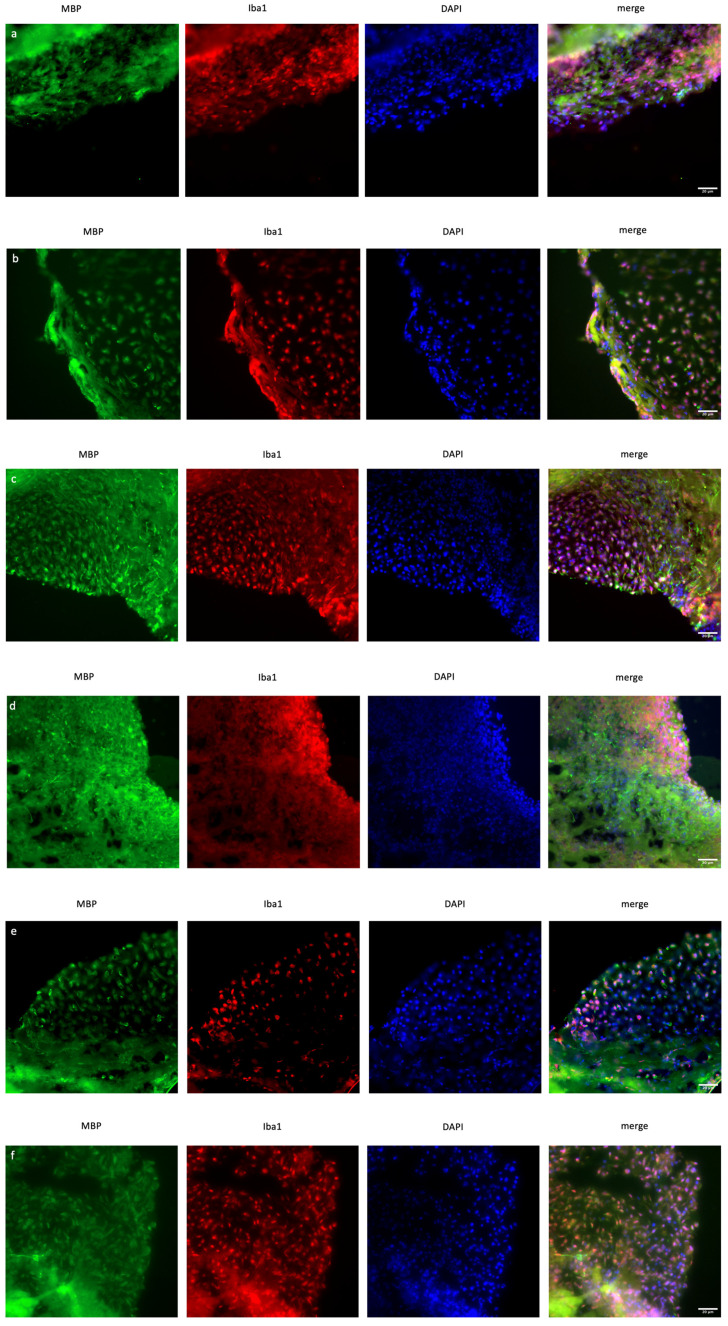

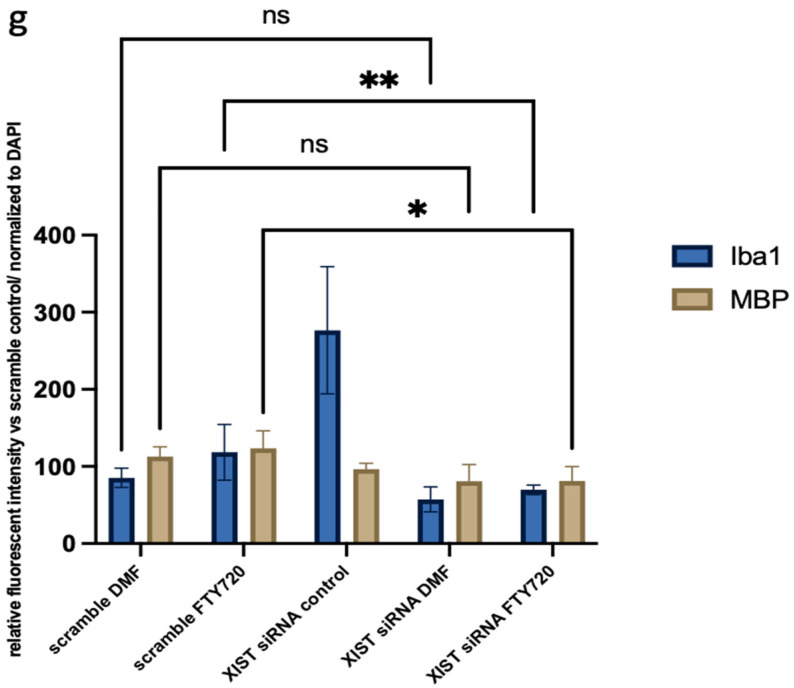
Immunofluorescence staining of scramble- and *XIST*-silenced organoid sections (**a**,**d**). Immunofluorescence staining of organoid sections obtained from scramble (**a**)- and *XIST*-siRNA (**b**)-transfected H9 hESCs. (**b**,**e**) Immunofluorescence staining of organoid sections obtained from scramble (**b**)- and *XIST*-siRNA (**e**)-transfected H9 hESCs after treatment with DMF. (**c**,**f**) Immunofluorescence staining of organoid sections obtained from scramble (**c**)- and *XIST*-siRNA (**f**)-transfected H9 hESCs after treatment with FTY720. (**g**) Calculation of the relative fluorescence intensity of MBP and microglia-specific (IBA1) antibodies. The results were compared with those of the scramble group and normalized to those of the DAPI group. Relative fluorescence intensity was calculated in tissue sections obtained from two independent experiments, and two technical replicates of the experiment for each sample were performed for each staining (*n* = 2). The results obtained from two independent experiments (*n* = 2) were imported into GraphPad, and two-way ANOVA was performed on these two independent experiments (ns = *p* > 0.05, * = *p* ≤ 0.05, ** = *p* ≤ 0.01, *** = *p* ≤ 0.001, and **** = *p* ≤ 0.0001). Blue: DAPI (nucleus), red: IBA1 (microglia), and green: MBP (myelination). The images were taken with a Leica DM6 B microscope at 40× magnification. Scale bars indicate 20 µm.

In organoid sections, ionized calcium-binding adapter molecule 1 (IBA1) expression was measured to determine the induction of inflammation in mature cerebral organoids. In *XIST*-silenced organoids, IBA1 protein expression was significantly greater than that in control scramble-siRNA-transfected organoids (Figure 4). In *XIST*-silenced organoids, microglial activation was altered in response to FTY720 but not DMF. The MBP protein was used as a marker to assess myelination. MBP staining was not significantly different between the *XIST*-silenced and control organoids. DMF did not affect myelination in *XIST*-silenced organoids. MBP levels responded differently to FTY720 treatment in *XIST*-silenced and control organoids (Figure 4). Thus, *XIST* controls microglial activation to reduce inflammation in LPS-treated cerebral organoids, but it does not affect myelination. The potential detrimental impact of the transfection reagent on organoid development was eliminated by inducing both myelination and inflammation in the organoids derived from untransfected control H9 hESCs (Figure S3).

### 2.5. Silencing of XIST Affects the Level of Alpha-Synuclein Secretion

**Figure 5:**
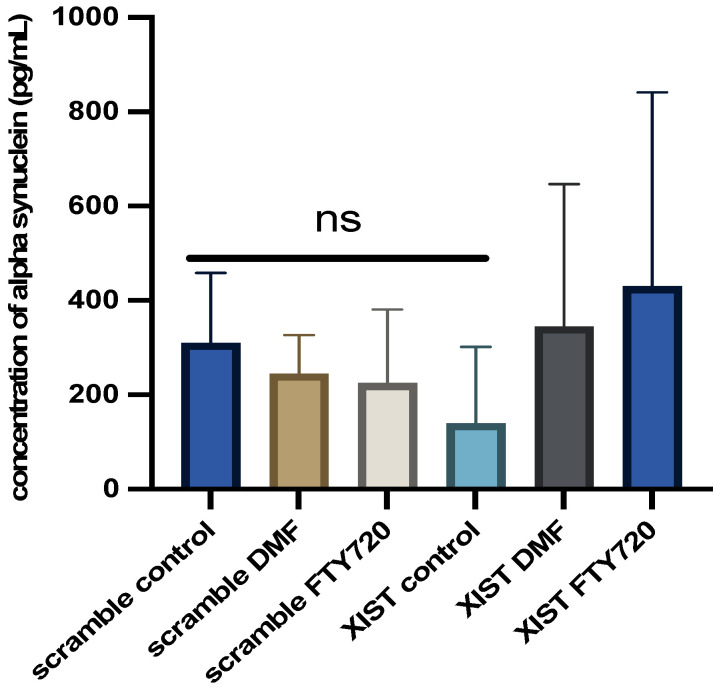
Determination of the differential effects of DMF and FTY720 treatment on alpha-synuclein protein expression in the presence of *XIST* silencing. Proteins were obtained from the organoid medium after 60 h of treatment with DMF and FTY720 or from untreated organoids, and the protein amount was calculated via the Human Alpha-Synuclein SimpleStep ELISA Kit. Two replicates of the experiment were performed for each sample. The results obtained from two independent experiments (*n* = 2) were imported into GraphPad, and two-way ANOVA was performed on these two independent experiments (ns = *p* > 0.05, * = *p* ≤ 0.05, ** = *p* ≤ 0.01, *** = *p* ≤ 0.001, and **** = *p* ≤ 0.0001).

Owing to its role in neuroinflammation, the alpha-synuclein level was also quantified in LPS-induced cerebral organoids with and without drug treatment. In *XIST*-silenced organoids, alpha-synuclein protein levels decreased from 300 pg/mL to 100 pg/mL. *XIST* silencing decreased the alpha-synuclein protein level in scramble-treated tissues, but neither DMF nor FTY720 did. Additionally, FTY720 and DMF restored alpha-synuclein levels to control levels in the organoids (Figure 5).

### 2.6. Silencing of XIST Caused Differential Expression of Genes Related to Neural Development and Morphogenesis in Human Cerebral Organoids

**Figure 6:**
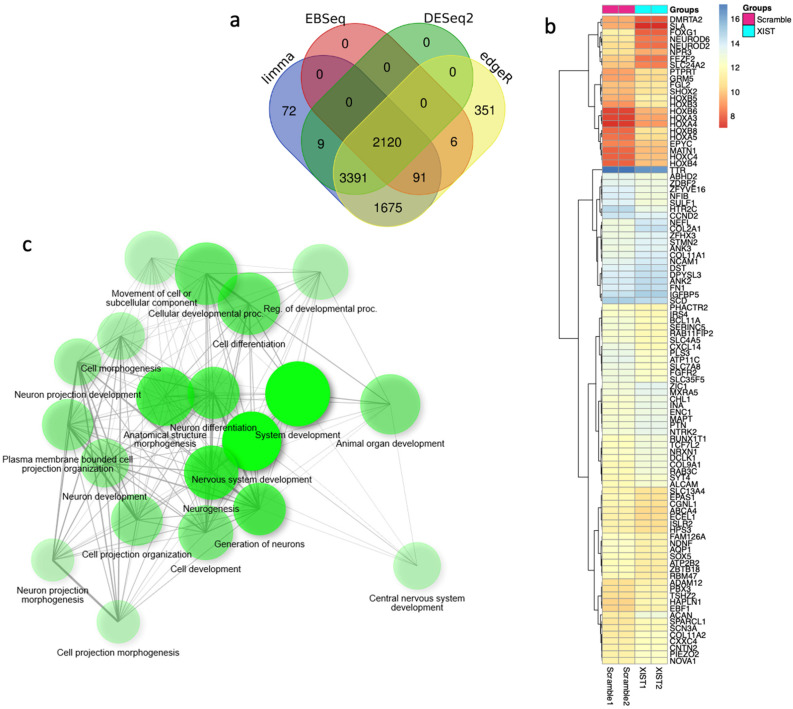
The key differentially expressed genes were identified via transcriptome analysis after *XIST* silencing. (**a**) Venn diagram of differentially expressed genes obtained via four methods: limma, EBSeq, DESeq2, and edgeR. (**b**) Heatmap of differentially expressed genes before and after *XIST* silencing. (**c**) Determination of differentially expressed genes involved in biological processes by GSEA after *XIST* silencing.

For a comprehensive analysis of the effects of *XIST* silencing, transcriptome analysis was performed, and the differentially expressed genes were determined. To identify more genes, four different analysis methods were used, and 2120 common genes were selected for further analysis (Figure 6a). The heatmap results revealed that *XIST* silencing increased the expression of 63 genes and decreased the expression of 36 genes significantly (Figure 6b). The GSEA results revealed that 20 DEGs were involved in development-related processes, 9 of which were related to neuronal differentiation (Figure 6b). Additionally, functional category analysis revealed that 102 DEGs were directly affected by the immune response (Table S4). Finally, downregulated and upregulated genes were evaluated separately via differential gene set enrichment analysis (GSEA) to determine their role in biological functions (Figure S4).

### 2.7. Silencing of XIST Did Not Significantly Affect Secretome Profiling Related to Immune Response and Myelination

For further confirmation of the comprehensive role of *XIST*, secretome analysis was also performed on both *XIST*-silenced and scramble organoids. Out of 96, 13 of secreted proteins were detected to be differentially expressed in the *XIST*-silenced organoids. Despite the inadequate number of secreted proteins, some of the differentially expressed ones such as COL11A1, COL2A1, and NES proteins belonging to “Extracellular Matrix-Related Protein” were detected, and they were also shown to express differentially at the transcriptome level (Table S5). Overall, the obtained data were not enough to conclude the role of *XIST* on immune- and myelination-related secretome profiling, so further future studies are required in this field.

## 3. Discussion

Although cerebral organoids cannot completely replicate the in vivo environment, they offer insightful analysis of brain development and disease models. Organoids lack peripheral immunological components such T and B cells, which are required for neuroinflammatory responses. Yet, microglia-like cells can proliferate within organoids. A malfunctioning vascular system restricts nutritional transmission, immune cell infiltration, and exchanges of systemic inflammatory signals, and extracellular matrix does not completely replicate the biochemical and mechanical characteristics of the in vivo brain, hence, influencing neuron–glia interactions required for myelination and immunological responses. These constraints call for more improvement of cerebral organoid models to increase their physiological relevance for studies on neuroinflammation and myelination disorders.

Studies have indicated the potential involvement of *XIST* in the predisposition of females to many neurodegenerative and autoimmune disorders, as well as cancer and cardiovascular diseases, in addition to its essential function in dose compensation in females [3,38]. Compared with males, females have a 2–4-fold greater predisposition to MS [12,39,40]. Immune response profiling, pregnancy, hormones, smoking, obesity, and genetic factors contribute to MS female bias [41]. *XIST* is a genetic factor that randomly inactivates one of the X chromosomes in females [42]. During XCI, *XIST* recruits particular proteins, including RNA-binding proteins, repressor proteins, heterochromatin and architectural proteins, and DNA methylases, which induce gene silencing, modify the chromatin environment, and reorganize the X chromosome [3,43,44]. In the AD mouse model and N2a cells, increased levels of *XIST* were observed in response to the oxidative stress induced by H_2_O_2_. Bioinformatics investigations revealed a binding site between miR-124 and ß-amyloid precursor protein-cleaving enzyme-1 (APP), whose dysregulation leads to increased Aβ synthesis and contributes to Alzheimer’s disease pathogenesis [45]. In the context of another autoimmune disease, systemic lupus erythematosus (SLE), the abnormal localization of *XIST* has been associated with changes in T cells among SLE patients, as well as alterations in *XIST* levels in SLE patients [38]. In a separate investigation, elevated serum levels of *XIST* were associated with the Unified Parkinson’s Disease Rating Scale in patients with Parkinson’s disease [46]. These studies indicate that *XIST* plays an indisputable role in the course of autoimmune and neurodegenerative diseases.

Moreover, 11–30% of human X-related genes escape *XIST* silencing and remain expressed to some extent [15]. Although the role of some of these genes in escaping *XIST* silencing in autoimmunity has been shown, considering the high percentage of these genes, more studies are needed to elucidate their role in autoimmunity and neuroinflammation and identify other escape genes that play roles in autoimmune diseases, especially MS [16]. Therefore, we investigated whether *XIST* might be an underlying factor in MS gender bias. To test our hypothesis, the *XIST* gene was silenced in H9 hESCs with *XIST*-siRNA, and up to a 20-fold decrease in the expression of *XIST* was confirmed via qRT-PCR (Figure S1). As shown by Cui and colleagues [47], there was a 3.7-fold increase in *FOXP3* gene expression in *XIST*-siRNA-transfected cells compared with MOCK-transfected cells (Figure S1). Since the transient effect of *XIST* silencing with *XIST*-siRNA was targeted in this study, no further silencing of *XIST* was evaluated in further steps. Additionally, the knockout of *XIST* was not preferred due to several disadvantages, including the observation that human stem cells devoid of *XIST* demonstrated a loss of differentiation, as evidenced via teratoma assays [48]. In fact, the conditional knockout of *XIST* leads to a greater development of larger gastrointestinal tumors under stress than does the wild type [49]. The impact of *XIST* deletion on the propensity for aggressive tumor growth and the impairment of stem cell differentiation capabilities was corroborated by a recent study [50]. Thus, human cerebral organoids were created from MOCK-, scramble-, and *XIST*-siRNA-transfected H9 cells via optimized protocols proposed by Lancaster and Knoblich [34]. The protocol by Madhavan and colleagues for generating myelinated organoids was integrated with the organoid generation process [51]. LPS treatment was administered before the application of DMTs to elicit an inflammatory response [52]. The daily morphological changes in the organoids were consistent with those reported in the literature, and the three biological groups had similar morphologies (Figure 1).

In the optimized protocol selected for organoid generation, some morphological changes at specific time points were indicated for subsequent differentiation. The first morphological sign of embryoid body formation is the shape and size of the body. Neural induction is initiated when embryoid bodies form circular structures with brightening and smooth edges and when the diameter of these bodies reaches 500–600 µm. Neuroepithelium formation was indicated by the observation of radial organization in the embryoid bodies. The third morphological indicator of differentiation is budding formation after the tissues are embedded in Matrigel [34]. Human cortical organoids have a characteristic lamination pattern in which progenitor cells are located inside the structure, whereas differentiated neuron cells are found in the outer layer of the structure [53]. Consistent with the literature, while neuronal stem cells were localized in the inner parts, excitatory neurons were mostly located on the outer surface of the cerebral organoids (Figure 2a,b). Furthermore, the presence of astrocytes and mature oligodendrocyte markers in the tissues confirmed the effective differentiation of neurons along with glial cells in the cerebral organoids (Figure 2c,d).

This study aimed primarily to elucidate the role of *XIST* in neuroinflammation and the processes of demyelination and remyelination, with a focus on neurodegenerative pathogenesis of MS. To test our hypothesis in cerebral organoids, the localization of oligodendrocytes, their maturation, and, finally, their myelination steps are crucial. Additionally, the precise localization of these particular cells within designated regions of the tissues is essential for the thorough identification of organoids. For this reason, tau expression was initially demonstrated in cerebral organoids to validate the presence of fully developed oligodendrocytes (Figure 2c,d). MBP protein expression subsequently occurred in the mature organoids (Figure 4). Nevertheless, the change in MBP protein expression could not be monitored during the organoid differentiation process because it was not possible to obtain high-quality organoid sections in the early stages. The protein expression levels of IL6 and IL10, as well as those of MBP and PLP1, were analyzed as indicators of inflammation and myelination, respectively, with and without *XIST* silencing. In LPS-induced cerebral organoids, long non-coding RNA *XIST* silencing altered *MBP* expression (Figure 3). *PLP1* and *MBP* are autoantigens that play a role in MS pathogenesis, and higher levels of MBP-specific autoreactive T cells are detected in the blood of MS patients [54,55]. Increased expression of inducible nitric oxide synthase (iNOS) and the proinflammatory cytokines IL-1β, IL-1α, IL-6, and TNF-α was reported in MBP-primed T cells isolated from female microglia but not male microglia, suggesting a sex bias in MS [56]. Our data demonstrate that *XIST* silencing leads to a notable increase in MBP expression, whereas PLP1 levels remain relatively stable. The absence of a corresponding increase in PLP1 expression suggests that *XIST*’s regulation of myelin components is selective and may involve a complex interaction between inflammatory signals and intrinsic oligodendrocyte differentiation programs. Our study demonstrated that *XIST* silencing modulated the expression of essential inflammatory cytokines, which are recognized for their role in oligodendrocyte maturation and myelination. The increased expression of *MBP* after *XIST* silencing in organoids could indicate the neuroprotective effect of *XIST* in MS pathogenesis due to the role of MBP in MS pathogenesis as an autoantigen. Since there is a gap in the literature explaining the relationship between myelination and *XIST*, the upregulation of *MBP* expression by *XIST* silencing could be a good starting point for understanding how *XIST* affects MS pathogenesis. This study demonstrated that siRNA-mediated silencing of *XIST* led to a temporary knockdown, a common characteristic of siRNA-based methods attributed to the degradation and dilution of siRNA molecules throughout organoid growth and cellular division. Transient silencing is beneficial for examining immediate or short-term effects; however, it may not adequately reflect the extended role of *XIST* in neuroinflammation, myelination, and therapeutic responses. In contrast, continuous or sustained silencing, achieved through methods such as CRISPR interference (CRISPRi), may offer more stable and prolonged inhibition of *XIST*, facilitating a more thorough understanding of its long-term effects during organoid maturation. To further understand the role of *XIST* in myelination, we recognize the need for future mechanistic research.

*XIST* silencing also affected the inflammatory response of *IL-10* and *IL-6* expression in cerebral organoids. In organoids from *XIST*-silenced H9 hESCs, *IL-10* and *IL-6* expression was increased 1.8-fold and 2.8-fold, respectively, compared with that in the scramble group (Figure 3). In contrast to some studies reporting the proinflammatory effect of *XIST* and increased *IL-6* levels in J774A.1 cells in response to LPS induction after *XIST* knockdown, the present study demonstrated dual (both pro- and anti-inflammatory) effects [57]. A recent study revealed that *XIST* activates the IL-6/STAT3 pathway to promote inflammation and cancer stem cell self-renewal [58]. Although some studies have shown that *XIST* plays a role in regulating inflammation, the exact role of *XIST* in regulating the inflammatory response and myelination processes has not been previously elucidated. A bioinformatics study reported the upregulation of *XIST* in MS, highlighting the importance of *XIST*-miR-326-HNRNPA1 in MS [25]. *XIST* alters immune responses by modulating the expression of pro-inflammatory cytokines in glial cells. Activated astrocytes exhibit *XIST* functioning as a competing endogenous RNA (ceRNA), which sponges specific microRNAs, resulting in increased expression of IL-1β, IL-6, and TNF-α, thereby exacerbating neuronal injury [24]. *XIST* also significantly influences the differentiation of Th17 cells that play a crucial role in the pathogenesis of MS by secreting IL-17 and other cytokines that promote neuroinflammation and demyelination. Strikingly, *XIST* functions as an inhibitor of Th17 cell differentiation. A recent study demonstrated that *XIST* inhibits Th17 differentiation via a microRNA-mediated mechanism. Specifically, *XIST* acts as a sponge for miR-377-3p, leading to the upregulation of the transcription factor ETS1, which negatively regulates Th17 lineage commitment [24]. So, *XIST* may function as a molecular scaffold or recruiter for chromatin remodeling complexes and transcription factors that modulate cytokine expression. The observed dual effect on IL-6 and IL-10 may be attributed to these interactions, indicating that *XIST*’s involvement in inflammation is context-dependent and closely associated with broader regulatory networks. The validation of these interactions through ChIP or similar methodologies is necessary. Considering the gap in the literature explaining the role of *XIST*, the differential expression of myelination- and inflammation-related genes after silencing *XIST* might be a good starting point to clarify the role of *XIST* in MS pathogenesis. However, further studies are needed to determine the underlying mechanisms of *XIST*-controlled inflammation or myelination.

To determine whether *XIST* affects the response to the two different disease-modifying drugs used in MS treatment, scramble- and *XIST*-silenced organoids were further treated with DMF or FTY720. DMF and FTY720 are two well-studied FDA-approved oral drugs with high efficacy against MS [59,60,61,62]. However, responses to different DMTs might be differentiated on the basis of several complex processes, such as the stage of the disease, the region affected, and age [63]. For this reason, the reasons behind the different responses to the treatments need to be evaluated to obtain better results in the treatment of MS. Considering these complex mechanisms, we also investigated the effect of *XIST* silencing on the response to two different DMTs, FTY720 and DMF.

Compared with scramble treatment, DMF treatment increased *MBP* and *PLP1* levels, but FTY720 decreased their expression (Figure 3). The differential expression of *IL-6* and *IL-10* in response to FTY720 and DMF treatment in *XIST*-silenced organoids highlight *the* dual role of *XIST* in inflammation at the transcriptional level (Figure 3). Therefore, further studies were conducted at the protein level to determine the ultimate effect of *XIST* silencing. IBA1 is a microglial marker that contributes to the phagocytosis process and reconstruction of the microglial cytoskeleton [64,65,66,67,68,69]. The immunofluorescence staining results demonstrated that the protein expression of IBA1 was significantly increased in *XIST*-silenced cerebral organoids, suggesting the potential role of *XIST* in microglial activation (Figure 2). Shenoda and colleagues [57] reported that *XIST* decreases the inflammatory response in J774A.1 female mouse macrophages. These authors reported that the anti-inflammatory response was correlated with alleviating the inflammatory response to dexamethasone. Thus, the anti-inflammatory role of *XIST* may be exerted through the regulation of microglial activation. Considering the anti-inflammatory role of both DMF and FTY720, a significant decrease in microglial activation was observed after treatment with DMF and FTY720 in *XIST*-silenced organoids [70,71]. Hence, *XIST* silencing changed the response to FTY720 but not DMF in terms of microglial activation. Unlike the inflammation-related protein IBA1, no substantial alteration in myelination was detected following the silencing of *XIST*. DMF treatment did not alter myelination in *XIST*-silenced tissues. However, MBP levels were altered in response to FTY720 treatment after *XIST* silencing but were insignificant. In summary, although *XIST* plays an anti-inflammatory role in LPS-induced cerebral organoids by controlling microglial activation, it does not affect the myelination process. Alpha-synuclein is another neuronal protein involved in the inflammation and pathogenesis of MS and neurodegenerative diseases [72,73,74]. The decreased concentration of alpha-synuclein in the medium of organoids obtained from *XIST*-siRNA-transfected cells further supports the anti-inflammatory role of *XIST* (Figure 5). Consistent with our findings, studies have demonstrated that the low levels of alpha-synuclein protein observed in MS patients support the protective role of *XIST* in neuroinflammation in addition to inflammation [72,75]. Although neither DMF nor FTY720 significantly changed the alpha-synuclein protein levels in control tissues, both drugs elevated alpha-synuclein protein levels in *XIST*-silenced organoids (Figure 5). As a result, the role of *XIST* in regulating alpha-synuclein protein levels was shown in the present study.

**Figure 7:**
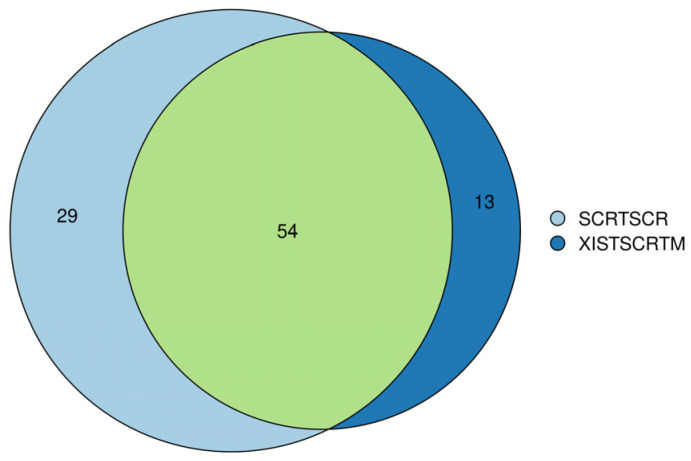
Venn diagram analysis of secretome analysis obtained from scramble (SCRTSCR) and *XIST*-silenced (*XIST*SCRTM) organoids. The image shows the compositions of overlap and unique proteins in the secretome of scramble and *XIST*-silenced organoids.

For the extensive analysis of *XIST* silencing, transcriptome analysis was performed, and differentially expressed genes were identified in human cerebral organoids (Figure 6a,b). GSEA was conducted to assess these findings. The biological process results indicate that these genes are involved in developmental processes, particularly those related to neuronal development. In the literature, the role of *XIST* in neuronal differentiation has been shown in recently published studies, but the exact mechanism has not been elucidated. In one study, *XIST* silencing caused neuronal differentiation in only female human pluripotent stem cells [76]. In addition to these recent findings, the present study revealed that *XIST* silencing increased the number of neuronal stem cells, excitatory neurons, astrocytes, and mature oligodendrocytes in human cerebral organoids (Figure 2). The transcriptome and bioinformatics analysis results obtained in this study also support neuronal differentiation and contribute to the successful characterization of organoids. Several neuronal-related transcripts were detected in the organoids obtained from both the *XIST*-silenced and control groups (Figure 6). In a patient-derived cell study published by Czermiński and Lawrence, the contribution of *XIST* to neural differentiation in neuronal stem cells was shown to be indirect in Down syndrome patients [77]. When we examined the differentially expressed genes that play a role in the immune response, we observed the upregulation of STAT6 in the *XIST*-silenced organoids. Another proinflammatory family member, CXCL14, was also upregulated. GFAP, an astrocyte and microglia activation marker, was also upregulated in *XIST*-silenced organoids, which was also supported by the immunofluorescence staining results (Table S4, Figure 2e) [78]. The downregulated and upregulated genes were differentiated in terms of their biological functions. Among the downregulated genes, cerebrospinal fluid circulation was the focus of our study because it is related to MS. The upregulated genes were also shown to be involved mainly in neuron-related processes (Figure S4). These results support the anti-inflammatory role of *XIST* in human cerebral organoids (Figure 4). The analysis of our secretome data further confirmed successful neuronal differentiation in human cerebral organoids (Figure 7, Tables S5 and S6). In the transcriptome data, a marker of astrocytes, GFAP, was identified, which was further corroborated by immunofluorescence staining. Additionally, another astrocyte marker, vimentin (Vim), was detected in the secretome of human cerebral organoids [79,80] (Table S5). Microtubule-associated protein 1B (MAP1B) is another protein detected in the secreted proteins of cerebral organoids (Table S5). This protein is distinctive in early neuronal development and important for the growth and guidance of axons [81]. In *XIST*-silenced tissues, the expression of Nestin (Nes), which is a neuronal stem cell marker, supports the role of *XIST* in neuronal development (Table S6). Another stem cell marker, SOX2, was also analyzed, revealing nearly doubled expression of this neuronal stem cell marker in the *XIST*-silenced tissues (Figure 2a,b, Table S5) [82]. Furthermore, certain secreted proteins identified in the secretome support indirectly our data on the anti-inflammatory role of *XIST*. For example, moesin (MSN), which is predominantly synthesized by microglia, is increased in the brains of AD patients, as characterized by *XIST*-silenced tissues (Tables S5 and S6) [83,84]. Poly (ADP-ribose)-polymerase-1 (PARP1) is another secreted protein that is differentially expressed in *XIST*-silenced tissues and is activated in MS and different neurodegenerative diseases, including Huntington’s disease and amyotrophic lateral sclerosis (Figure 7, Tables S5 and S6) [85]. Gene set enrichment analysis of differentially identified proteins in XIST-silenced organoid secretomes reveals convergent cellular pathologies rooted in the disruption of organelle organization, the collagen-associated extracellular matrix (ECM), and the endomembrane system (Table S6). These systems, though distinct, are intimately interconnected and collectively critical for maintaining neuronal homeostasis [86,87,88,89]. ECM alterations, particularly involving collagen degradation and remodeling, organelle dysfunction (mitochondrial and ER stress), and endomembrane/lysosomal disturbances, provide a more integrated picture of MS pathophysiology beyond pure immune cell attack. These factors contribute to the immune dysregulation by influencing immune cell metabolism and survival and, to the physical processes of demyelination and neurodegeneration, by determining how well CNS cells cope with stress and recover from injury in MS [90,91,92,93]. Each of these avenues is supported by emerging research, underscoring the importance of continued studies into the subcellular and microenvironmental underpinnings of MS. The observation of these proteins in the *XIST*-silenced tissues, but not in the scramble control, revealed the role of *XIST* (Tables S5 and S6).

Recent studies have shown that dysregulated lncRNAs such as MALAT1, NEAT1, and *XIST* contribute to MS pathogenesis [24,94,95,96,97]. MALAT1 dysregulation is linked to neurodegenerative illnesses such as AD, with research indicating that MALAT1 can directly regulate microglial activation and alter inflammatory responses. In contrast, NEAT1 is absolutely required for the development of nuclear paraspeckles and plays a key role in modulating the expression of inflammatory genes during cellular stress. NEAT1 expression variations have been associated to neurodegenerative illnesses such as amyotrophic lateral sclerosis and PD. While *XIST* may indirectly influence neuroinflammation by altering the inflammatory milieu, which affects myelination and neuronal integrity, MALAT1 and NEAT1 appear to play more direct roles in maintaining neuronal homeostasis and responding to cellular stress. These lncRNAs form a complementary network that impacts neuroinflammatory signaling and neurodegeneration, suggesting that they could be therapeutic targets, albeit through different mechanisms. Consequently, our results collectively demonstrate that *XIST* play a role in MS pathogenesis by regulating neuronal development, differentiation, and inflammation. However, further studies are needed to reveal the underlying mechanisms, binding partners, and upstream and downstream processes involved in these processes.

## 4. Materials and Methods

### 4.1. Feeder-Dependent Culture of H9 hESCs

The H9 female hESC cell line was purchased from WiCell (WA09) and cultured on mitotically inactive murine embryonic fibroblast (iMEF) feeder cells in hESC medium (20% knockout serum replacement (KOSR), 1% MEM nonessential amino acids (NEAA) solution, 55 µM 2-mercaptoethanol, and freshly added 4 ng/mL bFGF (10 µg/mL)) supplemented with 10 µM Y-27632 ROCK inhibitor (Stemcell™ Technologies, Köln, Germany), and the medium was replaced with 1 mL of fresh hESC medium daily until hESC colonies were observed on feeder cells. Later, colonies were transferred to growth factor-reduced Matrigel (Corning^®^ 354230, New York, NY, USA)-coated plates, and a standard passaging protocol of WiCell for H9 cell passage with EDTA was followed until enough cells were obtained.

### 4.2. Silencing of XIST by XIST-siRNA

To understand the role of *XIST* in neuroinflammatory pathogenesis within cerebral organoids, *XIST* lncRNA was silenced via the use of *XIST*-siRNA. To transfect *XIST*-siRNA into H9 cells, Lipofectamine™ RNAiMax Reagent (Invitrogen 13778075, Waltham, MA, USA) was used following the manufacturer’s instructions. The expression levels of *XIST* and specific *XIST*-related genes were subsequently analyzed to validate the transfection and silencing processes once before organoid generation. Detailed information about the *XIST* siRNA and universal negative control is shown in Table S1.

### 4.3. 3D Cerebral Organoid Generation from hESCs

Three biological groups of H9 cells were utilized for organoid generation: MOCK-transfected, *XIST*-siRNA-transfected, and scramble-transfected (universal negative siRNA) H9 cells. The organoids were produced by adhering to the optimal protocol [34]. When hESCs were ready to continue organoid generation, embryoid bodies (EBs) were generated. Before starting, the cells were treated with 20 µM ROCK inhibitor for 1 h at 37 °C to reduce cell death in the tissues. The cell colonies were collected by incubating them in 0.5 mM EDTA for 4 min and then Accutase solution for another 4 min, after which they were resuspended in low-bovine basic fluid (bFGF) hESC medium (20% knockout serum replacement (KOSR), 3% fetal bovine serum (FBS), 1% GlutaMAX, 1% NEAA, 0.05% 2-mercaptoethanol, and 4 ng/mL bFGF in DMEM-F12) containing ROCK inhibitor (1:100, final concentration 50 μM), and 9000 live cells/150 μL were plated on a low-attachment U-bottom 96-well plate and briefly centrifuged (at 200× *g*) for about 1 min to collect the cells at the bottom of the plate. A day after, half-volume of low-bFGF medium was directly added on EBs, and, for the upcoming days, the EBs were fed fresh low-bFGF medium every other day. During medium replacement, the medium on EBs was collected in a canonical tube to prevent loss of tissues during medium exchange. To generate primitive neuroepithelial tissues, EBs were transferred into neural induction medium (supplemented with 1% N2, 1% GlutaMAX, 1% MEM-NEAA, or 0.01% heparin in DMEM-F12) in low-attachment 24-well plates after the EBs reached 500–600 μm in diameter. The medium on the tissues was replaced with fresh neural induction medium every other day for 3 days. When radial organization was observed in neuroepithelial tissues, the tissues were embedded in 30 µL of Matrigel droplets. Throughout the Matrigel embedding procedure, all materials (gloves, Parafilm, and tweezers) were sterilized via UV exposure for a minimum duration of thirty minutes; during Matrigel embedding process, all materials used were pre-cold, and Matrigel embedding was performed on ice. In order to prevent unwanted polymerization of Matrigel outside, we tried to perform the embedding process as quick as possible. Dimples were formed via the use of Parafilm by placing it on a 200 µL tip tray. A 30 min incubation was conducted for the polymerization of Matrigel, after which Matrigel droplets containing neuroepithelial tissues were transferred to neural differentiation medium devoid of vitamin A [50% neurobasal, 0.5% N2 supplement, 1% B27 without vitamin A, 0.035% 2-mercaptoethanol (diluted 1:100 in DMEM-F12), and 1% penicillin/streptomycin in DMEM-F12]. For transferring tissues, dimples were turned inside out, and tissue-containing Matrigel droplets were sprayed with medium. When the spraying step was performed by one person, small pieces of Parafilm were cut; whereas, when it was performed by two people, bigger pieces were generated. The medium on the tissues was replaced with fresh neural differentiation medium without vitamin A for 48 h after transfer to Matrigel and then incubated for an additional 48 h. At the end of the incubation, the differentiation medium without vitamin A was replaced with differentiation medium with vitamin A, and the tissues were incubated for 48 h with this medium without agitation. When incubation was complete, the tissues were transferred to an orbital shaker (70 rpm), and a static culture of the tissues was started. After orbital shaker was switched on inside incubator, the humidifying chamber of incubator was more frequently checked to eliminate temperature increased caused by the shaker. The tissues were fed differentiation medium supplemented with vitamin A every 3–4 days until day 14 [34]. The optimized protocol was supported by another protocol proposed by Madhavan and colleagues to induce further neuronal differentiation and myelination [51]. On the 14th day, 20 ng/mL neurotropin 3 (NT3, Merck #GF308, Darmstadt, Germany) and 20 ng/mL brain-derived neurotropic factor (BDNF, Merck, #GF301) were added to the differentiation medium supplemented with vitamin A, and the tissues were fed differentiation medium supplemented with vitamin-A-containing NT3 and BDNF for 10 days. For the next 6 days, the tissues were fed differentiation medium supplemented with vitamin A without additional growth factors/supplements/small molecules. On day 32, the differentiation medium with vitamin A was supplemented with 10 ng/mL insulin growth factor (IGF1) and platelet-derived growth factor (PDGF, Merck, #GF142), and the tissues were incubated with this medium for 9 days. After that, the organoid tissues were fed 40 ng/mL 3,3′,5-triiodothyronine (T3, Sigma, #T2877,Darmstadt, Germany ) containing differentiation medium supplemented with vitamin A for 3 days, and 1 µM ketoconazole was added in addition to 40 ng/mL T3. The tissues were then incubated with differentiation medium supplemented with vitamin A, ketoconazole, and T3 for an additional 4 days. For the last 3 days before drug treatment, the organoids were fed only differentiation medium supplemented with vitamin A. Until the end of organoid growth, the medium was changed every 3–4 days after transfer to an orbital shaker without considering the composition of the medium. All growth factors and small molecules were freshly added into the differentiation medium, and the freeze and thaw processes, especially for growth factors and small molecules, were avoided. On the 56th day, after growth factor addition and organoid maturation, inflammation was induced in the organoids by treatment with 100 ng/mL lipopolysaccharide (LPS) for 24 h. The size and morphology of the organoids were followed daily during these differentiation processes, and organoids of approximately the same size and morphology were chosen for further analysis to eliminate bias due to variation in the organoids. At least 6–8 organoids from each group were selected randomly for further analysis of two independent biological replicates.

### 4.4. Treatment of Human Cerebral Organoids with Disease-Modifying Agents

Organoids derived from the *XIST*-siRNA of scramble-transfected H9 cells were exposed to 1.265 µM FTY720 or 11.5 µM DMF for 60 h. The concentration and duration of the treatments were optimized by pilot studies using cerebral organoids. The control organoids were cultured with only differentiation media containing vitamin A. After 60 h of incubation, the organoids were collected for further analysis.

### 4.5. Determination of Gene Expression via qRT-PCR

RNA isolation from H9 cells and organoids was performed via TransZol solution (TransGen Biotech ET101-01, Beijing, China). Total RNA was isolated from a pool of 6–8 whole organoids according to the manufacturer’s protocol. The RNA samples were subsequently used to synthesize cDNA via a cDNA synthesis kit following the manufacturer’s instructions (Abm G236, Richmond, BC, Canada). The expression levels of the genes tested in this study were quantified via qRT-PCR. For this purpose, the GoTaq qPCR Master enzyme was used, and standard cycling conditions provided by the manufacturers were applied. The details of the primers used for qRT-PCR are listed in Table S2. Four independent experiments were performed for each group (*n* = 4).

### 4.6. Immunofluorescence (IF) Staining of Organoid Tissues

Prior to the IF staining procedure, 6–8 organoids were randomly chosen from each group and rinsed with 1× PBS. The samples were subsequently fixed with 4% paraformaldehyde in 1× PBS for 15 min at 4 °C and washed with 1× PBS three times for at least 5 min each. The tissues were subsequently embedded in a 30% sucrose solution in 1× PBS for 24 h at 4 °C. The sucrose was then replaced with an optimum cutting temperature (OCT) solution, and the samples were stored at −80 °C until sectioning. Twenty-micrometer-thick tissue sections were obtained via a standard cryostat (Thermo Scientific HM525, Waltham, MA, USA) and preserved at −80 °C until the immunofluorescence staining procedure was performed. All IF staining procedures were conducted within a humidifying chamber. The sections stored at −80 °C were subjected to immunofluorescence staining by incubation with 0.25% Triton-X in 1× PBS for 10 min at room temperature. After incubation, the sections were washed twice with 1× PBS, with each washing step lasting a minimum of 5 min. The sections were subsequently incubated with 3% bovine serum albumin (BSA) in 0.025% Triton-X in 1× PBS for one hour at room temperature, followed by three washes with 1× PBS. Each washing step was conducted for a minimum duration of 5 min. The sections were subsequently incubated with fluorescently tagged primary antibodies overnight at 4 °C. The dilutions of the primary antibodies are presented in Table S3. Following overnight incubation with primary antibodies, the antibodies were removed from the sections, which were subsequently counterstained with 300 nM DAPI. The fluorescence images obtained with a Leica DM6 B microscope were imported into the ImageJ (version 1.53t) analysis program, and small areas on the sections were randomly selected for fluorescence intensity analysis to eliminate the possibility of regional bias during the evaluation of the results. The fluorescence intensity of the primary antibodies was normalized to the DAPI intensity. An overarching methodology was used for collecting and quantifying fluorescence images for immunofluorescence staining [98]. Two independent experiments were performed for each group (*n* = 2).

### 4.7. Determination of Alpha-Synuclein Levels via ELISA

The alpha-synuclein protein levels were determined in cultured human cerebral organoids via the Human Alpha-Synuclein SimpleStep ELISA Kit (Cambridge, UK). First, the culture medium of the 6 organoids was collected and centrifuged at 1000× *g* for 5 min at 4 °C twice to remove cell debris. The ELISA was conducted according to the manufacturer’s recommendations. Two independent experiments were performed for each group (*n* = 2).

### 4.8. Transcriptome Analysis

For transcriptome analysis, total RNA was isolated from six organoids transfected with either *XIST*-siRNA or scrambled shRNA via NucleoZol (Dueren, Germany) and quantified with a Qubit 4 fluorometer (Waltham, MA, USA). The Illumina Stranded Total RNA Prep and Ligation with Ribo-Zero Plus kits (San Diego, CA, USA) were used to construct an RNA sequencing library after the quality of the RNA was analyzed. Two technical replicates were used for the library construction of each sample. The constructed library was then sequenced via the Illumina NovaSeq 6000 platform (San Diego, CA, USA) to generate 70 million reads per sample.

### 4.9. Secretome Analysis

Each organoid culture medium sample was centrifuged to remove cellular debris, and the proteins were concentrated via StrataClean resin (Agilent Technologies, Santa Clara, CA, USA). After that, an on-bead protein digestion process was used as described elsewhere [99]. LC-MS/MS analysis was performed with a Dionex UltiMate 3000 RSLCnano UHPLC system (Waltham, MA, USA) to load the materials onto a Pepmap100 C18 trap column (5 µm, 0.3 × 5 mm; Thermo Scientific). Peptides were separated via an Easy Spray analytical column (PepMap RSLC C18, 75 µm × 25 cm, Thermo Scientific) with a linear gradient of 2–35% ACN/0.1% FA at 300 nL/min for 115 min. Under optimized conditions, an Orbitrap Fusion Tribrid mass spectrometer (Thermo Scientific) was used to analyze the isolated peptides [100]. RAW data were processed with MaxQuant default parameters [101]. The MS/MS spectra were compared to those of a UniProt human (UP000005640, https://www.uniprot.org/). A maximum of two missed cleavages were allowed with trypsin/P. Cysteine carbamidomethylation was fixed, but methionine oxidation and protein N-terminal acetylation were variable. A global false discovery rate (FDR) of 1% was applied for both protein and peptide identification. The match-between-runs and requantification of features were enabled. The identifications were considered valid if they had a minimum of 3 MS/MS counts and at least 2 razor peptides. A qualitative analysis of the secretome proteins in each group was performed manually.

### 4.10. Bioinformatic Analysis

The FastQC tool (version 0.11.7) was used to conduct quality control on the raw fastq data [102]. The readings were then trimmed via Trimmomatic software (version 0.39; [103]. The gene expression values were collected via the Kallisto tool (version 0.46.1) and the R package tximport (version 1.22.0) [104,105]. Owing to the small sample size, DEGs were identified via four different approaches: DESeq2 (version 1.40.2), edgeR (version 3.42.4), limma (version 3.56.2), and EBSeq (version 1.40.0) [106,107,108,109]. To reduce systematic disparities between samples, the data were normalized via the DESeq median ratio strategy before DESeq2 and EBSeq analysis and the trimmed mean of M values (TMM) methodology before edgeR and limma analysis. Before using limma, voom transformation was used [110]. To avoid the multiple testing problem, all *p* values were corrected via the Benjamini-Hochberg approach, and genes with adjusted *p* values of less than 5% were considered significant [111]. Venn diagrams were created to identify shared genes that were deemed relevant by all methodologies [106]. The DAVID Bioinformatics Resource was utilized to perform gene ontology (GO) and Kyoto Encyclopaedia of Genes and Genomes (KEGG) pathway analyses [112]. Gene set enrichment analysis (GSEA) was used to examine whether the discovered gene sets or biological pathways differed significantly between the relevant groups. The aforementioned analyses were carried out via the ShinyGo web program [113].

## 5. Conclusions

In conclusion, the findings of the present study demonstrated that silencing the *XIST* gene resulted in alterations in the expression of genes related to inflammation, myelination, and neuronal growth in cerebral organoids generated from H9 cells. As a result, this finding suggests that the *XIST* long non-coding RNA may be responsible for the sex discrepancy that is observed in Multiple Sclerosis and may be a promising target for therapeutic interventions. Furthermore, this research indicates that *XIST* may have additional regulatory functions in inflammation and neural development, distinct from its role in sex bias in neuroinflammation.

## Data Availability

The data that support the findings of this study are available at https://aguedutr-my.sharepoint.com/:f:/g/personal/nihan_aktas_agu_edu_tr/EgyCAbSgZF9AsHw9dcecL2UBNUM6ZttB1iKCvjTm4weWGQ?e=DaaTnK (accessed on 24 April 2025).

## Supplementary Materials

The following supporting information can be downloaded at: https://www.mdpi.com/article/10.3390/ncrna11030031/s1, Figure S1. Success of transfection check by expression of XIST and XIST-related genes, FOXP3 and Nanog by qRT-PCR compared to MOCK. The results obtained from two independent experiments were imported to Graphpad and statistical analysis was performed on these two independent experiments (n = 2) and, (ns = *p* > 0.05, * = *p* ≤ 0.05, ** = *p* ≤ 0.01, *** = *p* ≤ 0.001, **** = *p* ≤ 0.0001). Figure S2. Generation of myelinated and LPS-induced human cerebral organoids from MOCK H9 cells. (a) Seeding of MOCK H9 cells into U-bottom 96-well plate (day 0). (b) Observation of EBs obtained from MOCK H9 cells in U- bottom plates. (c) EBs obtained from MOCK H9 cells embedding into Matrigel when radial organization was observed in EBs 3 days after incubation with neural induction medium. (d) Culturing of EBs obtained from MOCK H9 cells in neural differentiation medium with vitamin A supplemented with growth factors and observation of small buddings in EBs. (e) Observation of myelinated and LPS induced human cerebral organoids obtained from MOCK H9 cells. The images were taken by Zeiss PrimoVert microscope with Dino Capture USB microscope camera. Scale bars indicate 500 µm. Figure S3. Immunofluorescence staining of organoid sections obtained from MOCK H9 hESCs. Blue: DAPI (nucleus), red: Iba1 (microglia), green: MBP (myelination). The images were taken by Leica DM6 B. Figure S4. GSEA analysis of differentially expressed genes. (a) Differentially downregulated genes enrichment bar plot involved in different biological processes. (b) Differentially upregulated genes enrichment bar plot involved in different biological processes. Table S1. Information about *XIST* siRNA and universal negative control. Table S2. Information about primers used in the study. Table S3. Details about primary antibodies used in immunofluorescence. Table S4. Determination of differentially expressed genes in biological processes by GSEA functional categories after XIST silencing. Table S5. Determination of differentially expressed secreted proteins after XIST silencing. Table S6. Gene set enrichment analysis of secreted proteins after XIST silencing.

## Abbreviations

The following abbreviations are used in this manuscript:

ACN	Acetonitrile
AD	Alzheimer’s disease
APP	ß-amyloid precursor protein-cleaving enzyme-1
BDNF	Brain-derived neurotropic factor
bFGF	Basic fibroblast growth factor
BSA	Bovine serum albumin
CD40L	Cluster of differentiation 40
ceRNA	Competitive endogenous RNA
CNS	Central nervous system
CXCL14	Chemokine (C-X-C motif) ligand 14
DAPI	4′,6-Diamidino-2-phenylindol
DAVID	The Database for Annotation, Visualization, and Integrated Discovery
DEGs	Differentially expressed genes
DMEM	Dulbecco’s modified Eagle’s medium
DMF	Dimethyl fumarate
DMTs	Disease-modifying therapies
EBs	Embryoid bodies
EDTA	Ethylenediaminetetraacetic acid
ELISA	Enzyme-linked immunosorbent assay
FA	Formic acid
FDA	Food and Drug Administration
FDR	False discovery rate
FOXP3	Forkhead box P3
FTY720	Fingolimod (Gilenya)
GFAP	Glial fibrillary acidic protein
GO	Gene ontology
GSEA	Gene set enrichment analysis
hESCs	Human embryonic stem cells
HNRNPA1	Heterogeneous nuclear ribonucleoprotein A
IBA1	Ionized calcium-binding adapter molecule 1
IF	Immunofluorescence
IGF1	Insulin growth factor
IL-10	Interleukin 10
IL-1α	Interleukin-1 alpha
IL-1β	Interleukin-1 beta
IL-6	Interleukin 6
iMEF	Mitotically inactive murine embryonic fibroblast
iNOS	Inducible nitric oxide synthase
IRAK-1	Interleukin-1 receptor-associated kinase 1
J774A.1	Mouse macrophage cells
KDM6A	Histone lysine demethylase 6A
KEGG	Kyoto Encyclopaedia of Genes and Genomes
KOSR	Knockout serum replacement
LC-MS/MS	Liquid chromatography–mass spectrometry
LPS	Lipopolysaccharide
MAP1B	Microtubule-associated protein 1B
MBP	Myelin basic protein
MEM	Minimal essential medium
MS	Multiple Sclerosis
MSN	Moesin
NANOG	Homeobox protein NANOG
NEAA	Nonessential amino acids
NT3	Neurotropin 3
OCT	Optimum cutting temperature
PARP1	Poly (ADP-ribose)-polymerase-1
PBS	Phosphate-buffered saline
PDGF	Platelet-derived growth factor
PLP1	Proteolipid protein 1
qRT-PCR	Quantitative reverse transcription polymerase chain reaction
ROCK	Rho-associated protein kinase
S1PRs	Sphingosine-1-phosphate receptors
SATB2	Special AT-rich sequence-binding protein 2
siRNA	Small inhibitory RNA
SLE	Systemic lupus erythematosus
SOX2	Sex-determining region Y-box 2
T3	3,3′,5-triiodothyronine
TAU	Tubulin associated unit
TLR7	Toll-like receptor 7
TMM	Trimmed mean of M
TNF	Tumor necrosis factor
TUBITAK	The Scientific and Technological Research Council of Türkiye
TUSEB	Health Institutes of Türkiye
UHPLC	Ultra-high-performance liquid chromatography
Vim	Vimentin
XCI	X chromosome inactivation
*XIST*	X-inactive specific transcript

## References

[1] Brown C.J., Hendrich B.D., Rupert J.L., Lafreniere R.G., Xing Y., Lawrence J., Willard H.F. (1992). The human XIST gene: Analysis of a 17 kb inactive X-specific RNA that contains conserved repeats and is highly localized within the nucleus. Cell.

[2] da Rocha S.T., Heard E. (2017). Novel players in X inactivation: Insights into Xist-mediated gene silencing and chromosome conformation. Nat. Struct. Mol. Biol..

[3] Li J., Ming Z., Yang L., Wang T., Liu G., Ma Q. (2022). Long noncoding RNA XIST: Mechanisms for X chromosome inactivation, roles in sex-biased diseases, and therapeutic opportunities. Genes Dis..

[4] Wang W., Min L., Qiu X., Wu X., Liu C., Ma J., Zhang D., Zhu L. (2021). Biological Function of Long Non-coding RNA (LncRNA) Xist. Front. Cell Dev. Biol..

[5] Siniscalchi C., Di Palo A., Russo A., Potenza N. (2022). The lncRNAs at X Chromosome Inactivation Center: Not Just a Matter of Sex Dosage Compensation. Int. J. Mol. Sci..

[6] He X., Yang L., Huang R., Lin L., Shen Y., Cheng L., Jin L., Wang S., Zhu R. (2020). Activation of CB2R with AM1241 ameliorates neurodegeneration via the Xist/miR-133b-3p/Pitx3 axis. J. Cell Physiol..

[7] Yue D., Guanqun G., Jingxin L., Sen S., Shuang L., Yan S., Minxue Z., Ping Y., Chong L., Zhuobo Z. (2020). Silencing of long noncoding RNA XIST attenuated Alzheimer’s disease-related BACE1 alteration through miR-124. Cell Biol. Int..

[8] Zhou Q., Zhang M.M., Liu M., Tan Z.G., Qin Q.L., Jiang Y.G. (2021). LncRNA XIST sponges miR-199a-3p to modulate the Sp1/LRRK2 signal pathway to accelerate Parkinson’s disease progression. Aging.

[9] Zhao Q., Lu F., Su Q., Liu Z., Xia X., Yan Z., Zhou F., Qin R. (2020). Knockdown of long noncoding RNA XIST mitigates the apoptosis and inflammatory injury of microglia cells after spinal cord injury through miR-27a/Smurf1 axis. Neurosci. Lett..

[10] Wang Y., Jiang F., Chen F., Zhang D., Wang J. (2022). LncRNA XIST Engages in Psoriasis via Sponging miR-338-5p to Regulate Keratinocyte Proliferation and Inflammation. Skin. Pharmacol. Physiol..

[11] Cohen J., Mathew A., Dourvetakis K.D., Sanchez-Guerrero E., Pangeni R.P., Gurusamy N., Aenlle K.K., Ravindran G., Twahir A., Isler D. (2024). Recent Research Trends in Neuroinflammatory and Neurodegenerative Disorders. Cells.

[12] D’Anca M., Buccellato F.R., Tartaglia G.M., Del Fabbro M., Muti P., Scarpini E., Galimberti D., Ghezzi L. (2023). Why Is Multiple Sclerosis More Frequent in Women? Role of the Immune System and of Oral and Gut Microbiota. Appl. Sci..

[13] Nytrova P., Dolezal O. (2022). Sex bias in multiple sclerosis and neuromyelitis optica spectrum disorders: How it influences clinical course, MRI parameters and prognosis. Front. Immunol..

[14] Klein S.L., Flanagan K.L. (2016). Sex differences in immune responses. Nat. Rev. Immunol..

[15] Krueger K., Lamenza F., Gu H., El-Hodiri H., Wester J., Oberdick J., Fischer A.J., Oghumu S. (2023). Sex differences in susceptibility to substance use disorder: Role for X chromosome inactivation and escape?. Mol. Cell Neurosci..

[16] Mousavi M.J., Mahmoudi M., Ghotloo S. (2020). Escape from X chromosome inactivation and female bias of autoimmune diseases. Mol. Med..

[17] Ngwa C., Misrani A., Manyam K.V., Xu Y., Qi S., Sharmeen R., McCullough L., Liu F. (2024). Escape of Kdm6a from X chromosome is detrimental to ischemic brains via IRF5 signaling. Res. Sq..

[18] Cipriano G.L., Schepici G., Mazzon E., Anchesi I. (2024). Multiple Sclerosis: Roles of miRNA, lcnRNA, and circRNA and Their Implications in Cellular Pathways. Int. J. Mol. Sci..

[19] Jalaiei A., Asadi M.R., Sabaie H., Dehghani H., Gharesouran J., Hussen B.M., Taheri M., Ghafouri-Fard S., Rezazadeh M. (2021). Long Non-Coding RNAs, Novel Offenders or Guardians in Multiple Sclerosis: A Scoping Review. Front. Immunol..

[20] Wang X., Wang C., Geng C., Zhao K. (2018). LncRNA XIST knockdown attenuates Aβ(25-35)-induced toxicity, oxidative stress, and apoptosis in primary cultured rat hippocampal neurons by targeting miR-132. Int. J. Clin. Exp. Pathol..

[21] Yan X.W., Liu H.J., Hong Y.X., Meng T., Du J., Chang C. (2022). lncRNA XIST induces Aβ accumulation and neuroinflammation by the epigenetic repression of NEP in Alzheimer’s disease. J. Neurogenet..

[22] Andrade F. (2024). Opinion: How does XIST promote sex bias in autoimmune diseases?. Front. Immunol..

[23] Yang J., Gong Z., Dong J., Bi H., Wang B., Du K., Zhang C., Chen L. (2023). lncRNA XIST inhibition promotes M2 polarization of microglial and aggravates the spinal cord injury via regulating miR-124-3p/IRF1 axis. Heliyon.

[24] Zeng M., Zhang T., Lin Y., Lin Y., Wu Z. (2023). The Common LncRNAs of Neuroinflammation-Related Diseases. Mol. Pharmacol..

[25] Ding Y., Li T., Yan X., Cui M., Wang C., Wang S., Zhang F., Zhang R. (2021). Identification of hub lncRNA ceRNAs in multiple sclerosis based on ceRNA mechanisms. Mol. Genet. Genom..

[26] Liu Z., Liao Q., Wen H., Zhang Y. (2021). Disease modifying therapies in relapsing-remitting multiple sclerosis: A systematic review and network meta-analysis. Autoimmun. Rev..

[27] Ferret-Sena V., Capela C., Macedo A., Salgado A.V., Derudas B., Staels B., Sena A. (2022). Fingolimod treatment modulates PPARgamma and CD36 gene expression in women with multiple sclerosis. Front. Mol. Neurosci..

[28] Roy R., Alotaibi A.A., Freedman M.S. (2021). Sphingosine 1-Phosphate Receptor Modulators for Multiple Sclerosis. CNS Drugs.

[29] Brennan M.S., Patel H., Allaire N., Thai A., Cullen P., Ryan S., Lukashev M., Bista P., Huang R., Rhodes K.J. (2016). Pharmacodynamics of Dimethyl Fumarate Are Tissue Specific and Involve NRF2-Dependent and -Independent Mechanisms. Antioxid. Redox Signal.

[30] Diebold M., Galli E., Kopf A., Sanderson N.S.R., Callegari I., Benkert P., Gonzalo Nunez N., Ingelfinger F., Herms S., Cichon S. (2022). High-dimensional immune profiling identifies a biomarker to monitor dimethyl fumarate response in multiple sclerosis. Proc. Natl. Acad. Sci. USA.

[31] Gross C.C., Schulte-Mecklenbeck A., Klinsing S., Posevitz-Fejfar A., Wiendl H., Klotz L. (2016). Dimethyl fumarate treatment alters circulating T helper cell subsets in multiple sclerosis. Neurol. Neuroimmunol. Neuroinflamm..

[32] Hojjati S., Ernerudh J., Vrethem M., Mellergard J., Raffetseder J. (2023). Dimethyl fumarate treatment in relapsing remitting MS changes the inflammatory CSF protein profile by a prominent decrease in T-helper 1 immunity. Mult. Scler. Relat. Disord..

[33] Wu Q., Wang Q., Mao G., Dowling C.A., Lundy S.K., Mao-Draayer Y. (2017). Dimethyl Fumarate Selectively Reduces Memory T Cells and Shifts the Balance between Th1/Th17 and Th2 in Multiple Sclerosis Patients. J. Immunol..

[34] Lancaster M.A., Knoblich J.A. (2014). Generation of cerebral organoids from human pluripotent stem cells. Nat. Protoc..

[35] Acar B., Aktas-Pepe N., Sen A. (2021). Optimization of myelination on human cerebral organoids for studying multiple sclerosis (MS). EuroBiotech J..

[36] Lancaster M.A., Renner M., Martin C.A., Wenzel D., Bicknell L.S., Hurles M.E., Homfray T., Penninger J.M., Jackson A.P., Knoblich J.A. (2013). Cerebral organoids model human brain development and microcephaly. Nature.

[37] Pagin M., Pernebrink M., Giubbolini S., Barone C., Sambruni G., Zhu Y., Chiara M., Ottolenghi S., Pavesi G., Wei C.L. (2021). Sox2 controls neural stem cell self-renewal through a Fos-centered gene regulatory network. Stem Cells.

[38] Syrett C.M., Paneru B., Sandoval-Heglund D., Wang J., Banerjee S., Sindhava V., Behrens E.M., Atchison M., Anguera M.C. (2019). Altered X-chromosome inactivation in T cells may promote sex-biased autoimmune diseases. JCI Insight.

[39] Boziki M., Theotokis P., Kesidou E., Karafoulidou E., Konstantinou C., Michailidou I., Bahar Y., Altintas A., Grigoriadis N. (2022). Sex, aging and immunity in multiple sclerosis and experimental autoimmune encephalomyelitis: An intriguing interaction. Front. Neurol..

[40] Kingwell E., Marriott J.J., Jetté N., Pringsheim T., Makhani N., Morrow S.A., Fisk J.D., Evans C., Béland S.G., Kulaga S. (2013). Incidence and prevalence of multiple sclerosis in Europe: A systematic review. BMC Neurol..

[41] Angeloni B., Bigi R., Bellucci G., Mechelli R., Ballerini C., Romano C., Morena E., Pellicciari G., Renie R., Rinaldi V. (2021). A Case of Double Standard: Sex Differences in Multiple Sclerosis Risk Factors. Int. J. Mol. Sci..

[42] Sun Z., Fan J., Wang Y. (2022). X-Chromosome Inactivation and Related Diseases. Genet. Res..

[43] Gupta K., Czerminski J.T., Lawrence J.B. (2024). Trisomy silencing by XIST: Translational prospects and challenges. Hum. Genet..

[44] Jacobson E.C., Pandya-Jones A., Plath K. (2022). A lifelong duty: How Xist maintains the inactive X chromosome. Curr. Opin. Genet. Dev..

[45] Modarresi F., Faghihi M.A., Patel N.S., Sahagan B.G., Wahlestedt C., Lopez-Toledano M.A. (2011). Knockdown of BACE1-AS Nonprotein-Coding Transcript Modulates Beta-Amyloid-Related Hippocampal Neurogenesis. Int. J. Alzheimer’s Dis..

[46] Zhang L., Li G., Lu D., Dai Q. (2022). High Expression of LncRNA XIST as an Index Helping to Diagnose Parkinson’s Disease. Neurophysiology.

[47] Cui X., Zhang C., Xu Z., Wang S., Li X., Stringer-Reasor E., Bae S., Zeng L., Zhao D., Liu R. (2022). Dual CRISPR interference and activation for targeted reactivation of X-linked endogenous FOXP3 in human breast cancer cells. Mol. Cancer.

[48] Anguera M.C., Sadreyev R., Zhang Z., Szanto A., Payer B., Sheridan S.D., Kwok S., Haggarty S.J., Sur M., Alvarez J. (2012). Molecular signatures of human induced pluripotent stem cells highlight sex differences and cancer genes. Cell Stem Cell.

[49] Yang L., Yildirim E., Kirby J.E., Press W., Lee J.T. (2020). Widespread organ tolerance to Xist loss and X reactivation except under chronic stress in the gut. Proc. Natl. Acad. Sci. USA.

[50] Richart L., Picod-Chedotel M.L., Wassef M., Macario M., Aflaki S., Salvador M.A., Hery T., Dauphin A., Wicinski J., Chevrier V. (2022). XIST loss impairs mammary stem cell differentiation and increases tumorigenicity through Mediator hyperactivation. Cell.

[51] Madhavan M., Nevin Z.S., Shick H.E., Garrison E., Clarkson-Paredes C., Karl M., Clayton B.L.L., Factor D.C., Allan K.C., Barbar L. (2018). Induction of myelinating oligodendrocytes in human cortical spheroids. Nat. Methods.

[52] Liu X., Huang Q., Li W., Yu J., Yu J., Yang Y., Song H., Liu Y., Niu X., Li W. (2024). The inhibitory impact of Schisandrin on inflammation and oxidative stress alleviates LPS-induced acute kidney injury. Biotechnol. Appl. Biochem..

[53] Chan W.K., Griffiths R., Price D.J., Mason J.O. (2020). Cerebral organoids as tools to identify the developmental roots of autism. Mol. Autism.

[54] Espino-Paisan L., Agudo-Jimenez T., Rosales-Martinez I., Lopez-Cotarelo P., Garcia-Martinez M.A., Dominguez-Mozo M.I., Perez-Perez S., Dieli-Crimi R., Comabella M., Urcelay E. (2020). A Polymorphism Within the MBP Gene Is Associated With a Higher Relapse Number in Male Patients of Multiple Sclerosis. Front. Immunol..

[55] Tejada-Simon M.V., Zang Y.C., Hong J., Rivera V.M., Zhang J.Z. (2003). Cross-reactivity with myelin basic protein and human herpesvirus-6 in multiple sclerosis. Ann. Neurol..

[56] Dasgupta S., Jana M., Liu X., Pahan K. (2005). Myelin basic protein-primed T cells of female but not male mice induce nitric-oxide synthase and proinflammatory cytokines in microglia: Implications for gender bias in multiple sclerosis. J. Biol. Chem..

[57] Shenoda B.B., Ramanathan S., Gupta R., Tian Y., Jean-Toussaint R., Alexander G.M., Addya S., Somarowthu S., Sacan A., Ajit S.K. (2021). Xist attenuates acute inflammatory response by female cells. Cell. Mol. Life Sci..

[58] Ma Y., Zhu Y., Shang L., Qiu Y., Shen N., Wang J., Adam T., Wei W., Song Q., Li J. (2023). LncRNA XIST regulates breast cancer stem cells by activating proinflammatory IL-6/STAT3 signaling. Oncogene.

[59] Lorscheider J., Benkert P., Lienert C., Hanni P., Derfuss T., Kuhle J., Kappos L., Yaldizli O. (2021). Comparative analysis of dimethyl fumarate and fingolimod in relapsing-remitting multiple sclerosis. J. Neurol..

[60] Masjedi S.S., Etemadifar M., Zadeh N.M., Afzali M. (2021). Assessment of fingolimod versus dimethyl fumarate for the treatment of multiple sclerosis; a 24-month follow-up study. Am. J. Clin. Exp. Immunol..

[61] Prosperini L., Lucchini M., Haggiag S., Bellantonio P., Bianco A., Buscarinu M.C., Buttari F., Centonze D., Cortese A., De Giglio L. (2018). Fingolimod vs dimethyl fumarate in multiple sclerosis: A real-world propensity score-matched study. Neurology.

[62] Zhu C., Kalincik T., Horakova D., Zhou Z., Buzzard K., Skibina O., Alroughani R., Izquierdo G., Eichau S., Kuhle J. (2023). Comparison Between Dimethyl Fumarate, Fingolimod, and Ocrelizumab After Natalizumab Cessation. JAMA Neurol..

[63] Engelhardt B., Comabella M., Chan A. (2022). Multiple sclerosis: Immunopathological heterogeneity and its implications. Eur. J. Immunol..

[64] Guerrero B.L., Sicotte N.L. (2020). Microglia in Multiple Sclerosis: Friend or Foe?. Front. Immunol..

[65] Jurga A.M., Paleczna M., Kadluczka J., Kuter K.Z. (2021). Beyond the GFAP-Astrocyte Protein Markers in the Brain. Biomolecules.

[66] Arcuri C., Mecca C., Bianchi R., Giambanco I., Donato R. (2017). The Pathophysiological Role of Microglia in Dynamic Surveillance, Phagocytosis and Structural Remodeling of the Developing CNS. Front. Mol. Neurosci..

[67] Qin J., Ma Z., Chen X., Shu S. (2023). Microglia activation in central nervous system disorders: A review of recent mechanistic investigations and development efforts. Front. Neurol..

[68] Fleiss B., Van Steenwinckel J., Bokobza C., Shearer I.K., Ross-Munro E., Gressens P. (2021). Microglia-Mediated Neurodegeneration in Perinatal Brain Injuries. Biomolecules.

[69] Cornell J., Salinas S., Huang H.Y., Zhou M. (2022). Microglia regulation of synaptic plasticity and learning and memory. Neural Regen. Res..

[70] Lee Y.S., Gupta D.P., Park S.H., Yang H.J., Song G.J. (2021). Anti-Inflammatory Effects of Dimethyl Fumarate in Microglia via an Autophagy Dependent Pathway. Front. Pharmacol..

[71] Li S., Sakurai K., Ohgidani M., Kato T.A., Hikida T. (2023). Ameliorative effects of Fingolimod (FTY720) on microglial activation and psychosis-related behavior in short term cuprizone exposed mice. Mol. Brain.

[72] Bilge N., Simsek F., Yevgi R., Ceylan M., Askin S. (2020). Low serum Alpha-SYNUCLEIN and oligomer Alpha-SYNUCLEIN levels in multiple sclerosis patients. J. Neuroimmunol..

[73] Machado-Santos J., Saji E., Troscher A.R., Paunovic M., Liblau R., Gabriely G., Bien C.G., Bauer J., Lassmann H. (2018). The compartmentalized inflammatory response in the multiple sclerosis brain is composed of tissue-resident CD8+ T lymphocytes and B cells. Brain.

[74] Ettle B., Kuhbandner K., Jorg S., Hoffmann A., Winkler J., Linker R.A. (2016). alpha-Synuclein deficiency promotes neuroinflammation by increasing Th1 cell-mediated immune responses. J. Neuroinflamm..

[75] Mejia M., Rodriguez-Leyva I., Cortes-Enriquez F., Chi-Ahumada E., Portales-Perez D.P., Macias-Islas M.A., Jimenez-Capdeville M.E. (2019). Low levels of alpha-synuclein in peripheral tissues are related to clinical relapse in relapsing-remitting multiple sclerosis: A pilot cross-sectional study. J. Neurol. Sci..

[76] Motosugi N., Sugiyama A., Okada C., Otomo A., Umezawa A., Akutsu H., Hadano S., Fukuda A. (2022). De-erosion of X chromosome dosage compensation by the editing of XIST regulatory regions restores the differentiation potential in hPSCs. Cell Rep. Methods.

[77] Czerminski J.T., Lawrence J.B. (2020). Silencing Trisomy 21 with XIST in Neural Stem Cells Promotes Neuronal Differentiation. Dev. Cell.

[78] Azzolini F., Gilio L., Pavone L., Iezzi E., Dolcetti E., Bruno A., Buttari F., Musella A., Mandolesi G., Guadalupi L. (2022). Neuroinflammation Is Associated with GFAP and sTREM2 Levels in Multiple Sclerosis. Biomolecules.

[79] Garcia A., Masot J., Franco A., Gazquez A., Redondo E. (2014). Immunohistochemical evaluation of the goat forestomach during prenatal development. J. Vet. Sci..

[80] Luo H., Wu X.Q., Zhao M., Wang Q., Jiang G.P., Cai W.J., Luo M.Y. (2017). Expression of vimentin and glial fibrillary acidic protein in central nervous system development of rats. Asian Pac. J. Trop. Med..

[81] Tortosa E., Montenegro-Venegas C., Benoist M., Hartel S., Gonzalez-Billault C., Esteban J.A., Avila J. (2011). Microtubule-associated protein 1B (MAP1B) is required for dendritic spine development and synaptic maturation. J. Biol. Chem..

[82] Baba Y., Onishi-Sakamoto S., Ide K., Nishifuji K. (2022). Nestin is a marker of unipotent embryonic and adult progenitors differentiating into an epithelial cell lineage of the hair follicles. Sci. Rep..

[83] Du Y., Bradshaw W.J., Leisner T.M., Annor-Gyamfi J.K., Qian K., Bashore F.M., Sikdar A., Nwogbo F.O., Ivanov A.A., Frye S.V. (2023). Discovery of FERM domain protein–protein interaction inhibitors for MSN and CD44 as a potential therapeutic approach for Alzheimer’s disease. J. Biol. Chem..

[84] Rayaprolu S., Gao T., Xiao H., Ramesha S., Weinstock L.D., Shah J., Duong D.M., Dammer E.B., Webster J.A., Lah J.J. (2020). Flow-cytometric microglial sorting coupled with quantitative proteomics identifies moesin as a highly-abundant microglial protein with relevance to Alzheimer’s disease. Mol. Neurodegener..

[85] Mao K., Zhang G. (2022). The role of PARP1 in neurodegenerative diseases and aging. FEBS J..

[86] van Dis V., Kuijpers M., Haasdijk E.D., Teuling E., Oakes S.A., Hoogenraad C.C., Jaarsma D. (2014). Golgi fragmentation precedes neuromuscular denervation and is associated with endosome abnormalities in SOD1-ALS mouse motor neurons. Acta Neuropathol. Commun..

[87] Wareham L.K., Baratta R.O., Del Buono B.J., Schlumpf E., Calkins D.J. (2024). Collagen in the central nervous system: Contributions to neurodegeneration and promise as a therapeutic target. Mol. Neurodegener..

[88] Cescon M., Chen P., Castagnaro S., Gregorio I., Bonaldo P. (2016). Lack of collagen VI promotes neurodegeneration by impairing autophagy and inducing apoptosis during aging. Aging.

[89] Chapman M.A., Sorg B.A. (2024). A Systematic Review of Extracellular Matrix-Related Alterations in Parkinson’s Disease. Brain Sci..

[90] Mohan H., Krumbholz M., Sharma R., Eisele S., Junker A., Sixt M., Newcombe J., Wekerle H., Hohlfeld R., Lassmann H. (2010). Extracellular matrix in multiple sclerosis lesions: Fibrillar collagens, biglycan and decorin are upregulated and associated with infiltrating immune cells. Brain Pathol..

[91] van den Berg R., Hoogenraad C.C., Hintzen R.Q. (2017). Axonal transport deficits in multiple sclerosis: Spiraling into the abyss. Acta Neuropathol..

[92] Misrielal C., Mauthe M., Reggiori F., Eggen B.J.L. (2020). Autophagy in Multiple Sclerosis: Two Sides of the Same Coin. Front. Cell. Neurosci..

[93] Delic S., Miletic Drakulic S., Stepovic M., Milosavljevic J., Kovacevic Dimitrijevic M., Jovanovic K., Marinkovic I., Tepavcevic M., Janicijevic N., Mitrovic A. (2025). The Connection Between Oxidative Stress, Mitochondrial Dysfunction, Iron Metabolism and Microglia in Multiple Sclerosis: A Narrative Review. NeuroSci.

[94] Sun D., Yu Z., Fang X., Liu M., Pu Y., Shao Q., Wang D., Zhao X., Huang A., Xiang Z. (2017). LncRNA GAS5 inhibits microglial M2 polarization and exacerbates demyelination. EMBO Rep..

[95] Masoumi F., Ghorbani S., Talebi F., Branton W.G., Rajaei S., Power C., Noorbakhsh F. (2019). Malat1 long noncoding RNA regulates inflammation and leukocyte differentiation in experimental autoimmune encephalomyelitis. J. Neuroimmunol..

[96] Dastmalchi R., Ghafouri-Fard S., Omrani M.D., Mazdeh M., Sayad A., Taheri M. (2018). Dysregulation of long non-coding RNA profile in peripheral blood of multiple sclerosis patients. Mult. Scler. Relat. Disord..

[97] Fenoglio C., Oldoni E., Serpente M., De Riz M.A., Arcaro M., D’Anca M., Pietroboni A.M., Calvi A., Lecchi E., Goris A. (2018). LncRNAs expression profile in peripheral blood mononuclear cells from multiple sclerosis patients. J. Neuroimmunol..

[98] Shihan M.H., Novo S.G., Le Marchand S.J., Wang Y., Duncan M.K. (2021). A simple method for quantitating confocal fluorescent images. Biochem. Biophys. Rep..

[99] Hammond D.E., Kumar J.D., Raymond L., Simpson D.M., Beynon R.J., Dockray G.J., Varro A. (2018). Stable Isotope Dynamic Labeling of Secretomes (SIDLS) Identifies Authentic Secretory Proteins Released by Cancer and Stromal Cells. Mol. Cell. Proteom..

[100] Azizoglu Z.B., Babayeva R., Haskologlu Z.S., Acar M.B., Ayaz-Guner S., Okus F.Z., Alsavaf M.B., Can S., Basaran K.E., Canatan M.F. (2024). DIAPH1-Deficiency is Associated with Major T, NK and ILC Defects in Humans. J. Clin. Immunol..

[101] Tyanova S., Temu T., Cox J. (2016). The MaxQuant computational platform for mass spectrometry-based shotgun proteomics. Nat. Protoc..

[102] Andrews S. (2010). FastQC: A Quality Control Tool for High Throughput Sequence Data.

[103] Bolger A.M., Lohse M., Usadel B. (2014). Trimmomatic: A flexible trimmer for Illumina sequence data. Bioinformatics.

[104] Bray N.L., Pimentel H., Melsted P., Pachter L. (2016). Near-optimal probabilistic RNA-seq quantification. Nat. Biotechnol..

[105] Soneson C., Love M.I., Robinson M.D. (2015). Differential analyses for RNA-seq: Transcript-level estimates improve gene-level inferences. F1000Research.

[106] Love M.I., Huber W., Anders S. (2014). Moderated estimation of fold change and dispersion for RNA-seq data with DESeq2. Genome Biol..

[107] Robinson M.D., McCarthy D.J., Smyth G.K. (2010). edgeR: A Bioconductor package for differential expression analysis of digital gene expression data. Bioinformatics.

[108] Ritchie M.E., Phipson B., Wu D., Hu Y., Law C.W., Shi W., Smyth G.K. (2015). limma powers differential expression analyses for RNA-sequencing and microarray studies. Nucleic Acids Res..

[109] Leng N., Dawson J.A., Thomson J.A., Ruotti V., Rissman A.I., Smits B.M., Haag J.D., Gould M.N., Stewart R.M., Kendziorski C. (2013). EBSeq: An empirical Bayes hierarchical model for inference in RNA-seq experiments. Bioinformatics.

[110] Law C.W., Chen Y., Shi W., Smyth G.K. (2014). voom: Precision weights unlock linear model analysis tools for RNA-seq read counts. Genome Biol..

[111] Benjamini Y., Hochberg Y. (1995). Controlling the False Discovery Rate: A Practical and Powerful Approach to Multiple Testing. J. R. Stat. Soc. Ser. B Methodol..

[112] Dennis G., Sherman B.T., Hosack D.A., Yang J., Gao W., Lane H.C., Lempicki R.A. (2003). DAVID: Database for Annotation, Visualization, and Integrated Discovery. Genome Biol..

[113] Ge S.X., Jung D., Yao R. (2020). ShinyGO: A graphical gene-set enrichment tool for animals and plants. Bioinformatics.

