# Supplementary material for: Role of Long Non-Coding RNA X-Inactive-Specific Transcript (XIST) in Neuroinflammation and Myelination: Insights from Cerebral Organoids and Implications for Multiple Sclerosis
Source: Noncoding RNA. 2025 Apr 29;11(3):31. doi: 10.3390/ncrna11030031 (PMC12101413; doi:10.3390/ncrna11030031)
Supplement: Supplementary file 1 [file ncrna-11-00031-s001.zip › ncrna-3539838-supplementary.pdf]

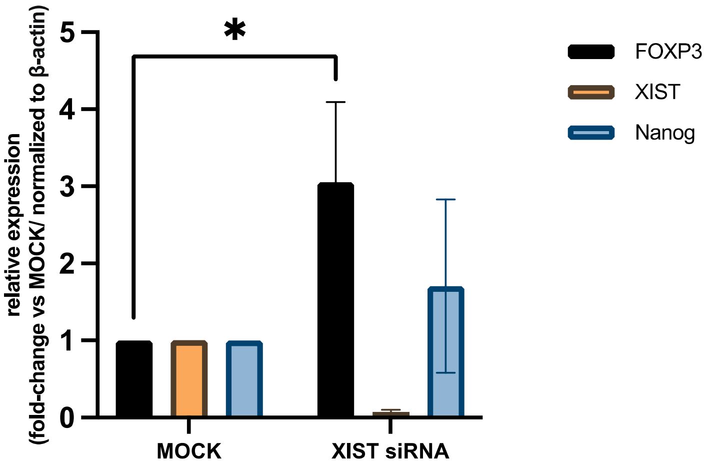

Figure S1. Success of transfection check by expression of XIST and XIST-related genes, FOXP3 and Nanog by qRT-PCR compared to MOCK. The results obtained from two independent experiments were imported to Graphpad and statistical analysis was performed on these two independent experiments (n=2) and, (ns=P>0.05, *= P ≤ 0.05, **= P ≤ 0.01, ***= P ≤ 0.001, ****= P ≤ 0.0001).

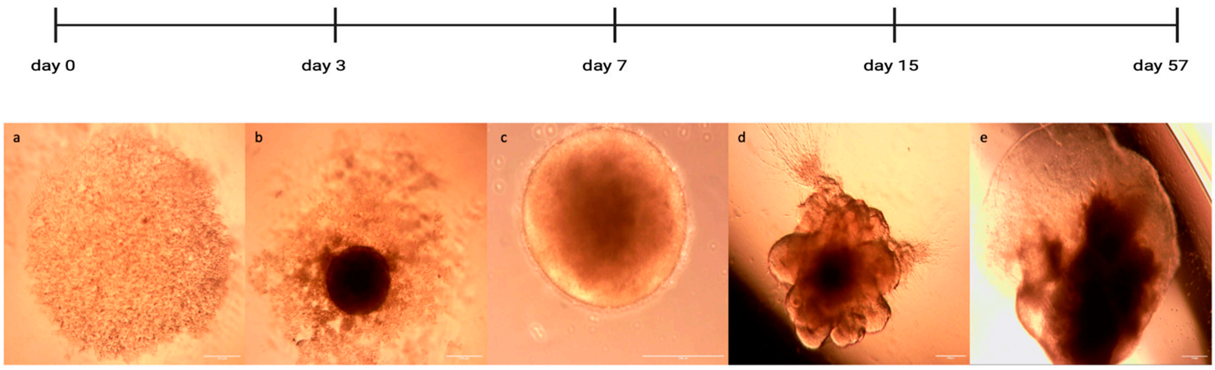

Figure S2. Generation of myelinated and LPS-induced human cerebral organoids from MOCK H9 cells. (a) Seeding of MOCK H9 cells into U-bottom 96-well plate (day 0). (b) Observation of EBs obtained from MOCK H9 cells in U- bottom plates. (c) EBs obtained from MOCK H9 cells embedding into Matrigel when radial organization was observed in EBs 3 days after incubation with neural induction medium. (d) Culturing of EBs obtained from MOCK H9 cells in neural differentiation medium with vitamin A supplemented with growth factors and observation of small buddings in EBs. (e) Observation of myelinated and LPS induced human cerebral organoids obtained from MOCK H9 cells. The images were taken by Zeiss PrimoVert microscope with Dino Capture USB microscope camera. Scale bars indicate 500 µm.

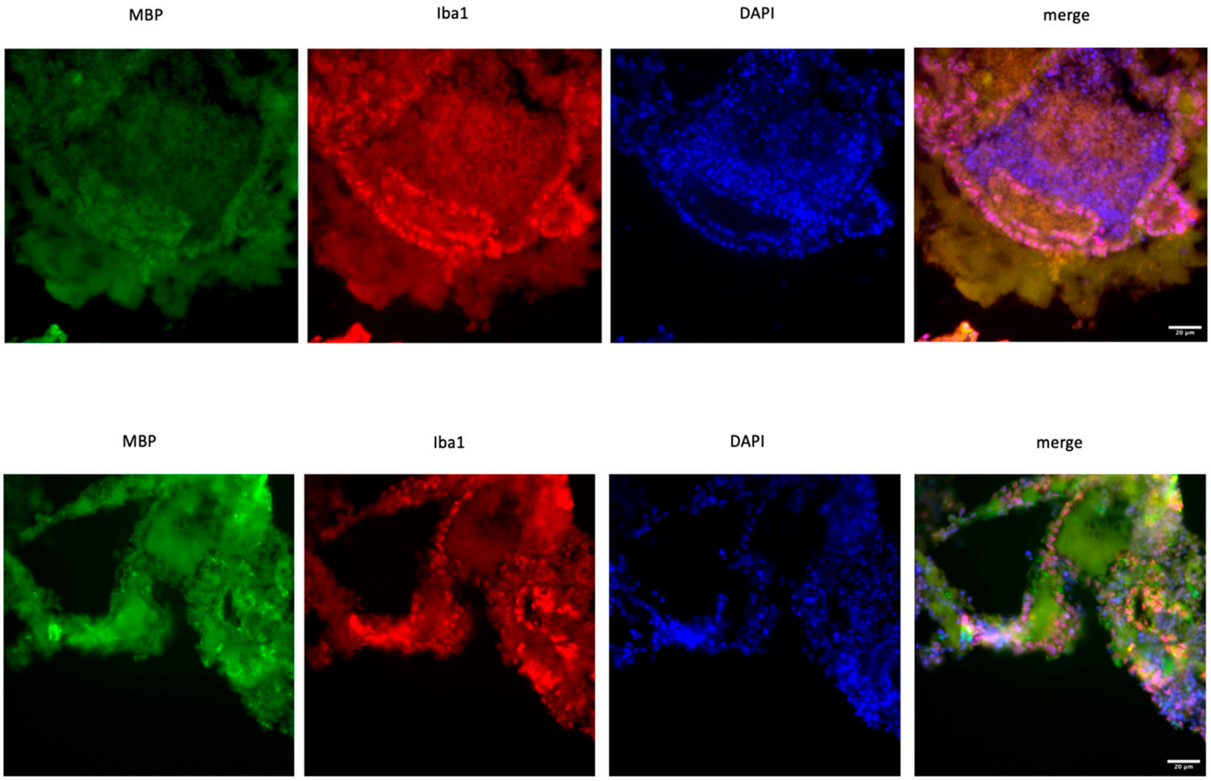

Figure S3. Immunofluorescence staining of organoid sections obtained from MOCK H9 hESCs. Blue: DAPI (nucleus), red: Iba1 (microglia), green: MBP (myelination). The images were taken by Leica DM6 B.

Table S1. Information about *XIST* siRNA and universal negative control.

Oligo Name	Sequence (5'-3')	Tm °
XIST siRNA	CUAAGGUGCAGGGCUUUAAA [dT][dT]	59.4
siRNA Universal Negative Control	UUUAAGCCCUGCACCUUAG [dT][dT]	60.8

Table S2. Information about primers used in the study

Code	Gene Name	Sequence (5'-3')	Tm °
NM_014009	FOXP3_F	GTGGCCCGGATGTGAGAAG	62.00
	FOXP3_R	GGAGCCCTTGTCGGATGATG	
NM_024865	NANOG_F	CTCCAACATCCTGAACCTCAGC	60.00
	NANOG_R	CGTCACACCATTGCTATTCTTCG	

NR_001564	XIST_F	GTAGGTGTGCTGATAACCAAGGC	60.00
	XIST_R	GGGAAAGGAAGATTGAGGGTGG	
NM_001101	ACTB_F	GCCGCCAGCTCACCAT	59.00
	ACTB_R	GATGCCTCTCTTGCTCTGGG	
NM_000600	IL6_F	ACTCACCTCTTCAGAACGAATTG	59.00
	IL6_R	CCATCTTTGGAAGGTTCAGGTTG	
NM_000533	PLP1_F	GAAAGCCCTTTTCATTGCAGGA	56.00
	PLP1_R	GGCTAGTCTGCTTTGTGGCT	
NM_002385	MBP_F	TCGGCTCACAAGGGATTCAAG	51.00
	MBP_R	TGATCCAGAGCGACTATCTCTTC	
NM_014009	IL10_F	TCTCCGAGATGCCTTCAGCAGA	51.00
	IL10_R	TCAGACAAGGCTTGGCAACCCA	

Table S3. Details about primary antibodies used in immunofluorescence.

Name of antibody	Fluorescent Tag	Catalog Number	Dilution (in 3 % BSA in 0.025 % Triton-X in 1X PBS)
anti-MBP antibody	Alexa Fluor ® 488	sc-271524	1:200
Iba1 antibody	Alexa Fluor ® 594	sc-32725	1:150
SATB2	Alexa Fluor ® 647	sc-518006	1:100
Anti-SOX2	Alexa Fluor ® 488	sc-365823	1:100
Anti-GFAP	Alexa Fluor ® 594	sc-33673	1:100
Tau	Alexa Fluor ® 488	sc-390476	1:100

Table S4. Determination of differentially expressed genes in biological processes by GSEA functional categories after XIST silencing.

GO_functional_categories.xlsx
https://aguedutr-my.sharepoint.com/:x:/g/personal/nihan_aktas_agu_edu_tr/ER9WJInzKkZFtYcXflC-4_EBmbhVYeefzOK51GTiIlK9xQ?e=aoT9kM&wdLOR=cE26B092B-D228-4C3B-8976-1EBECCD086E5

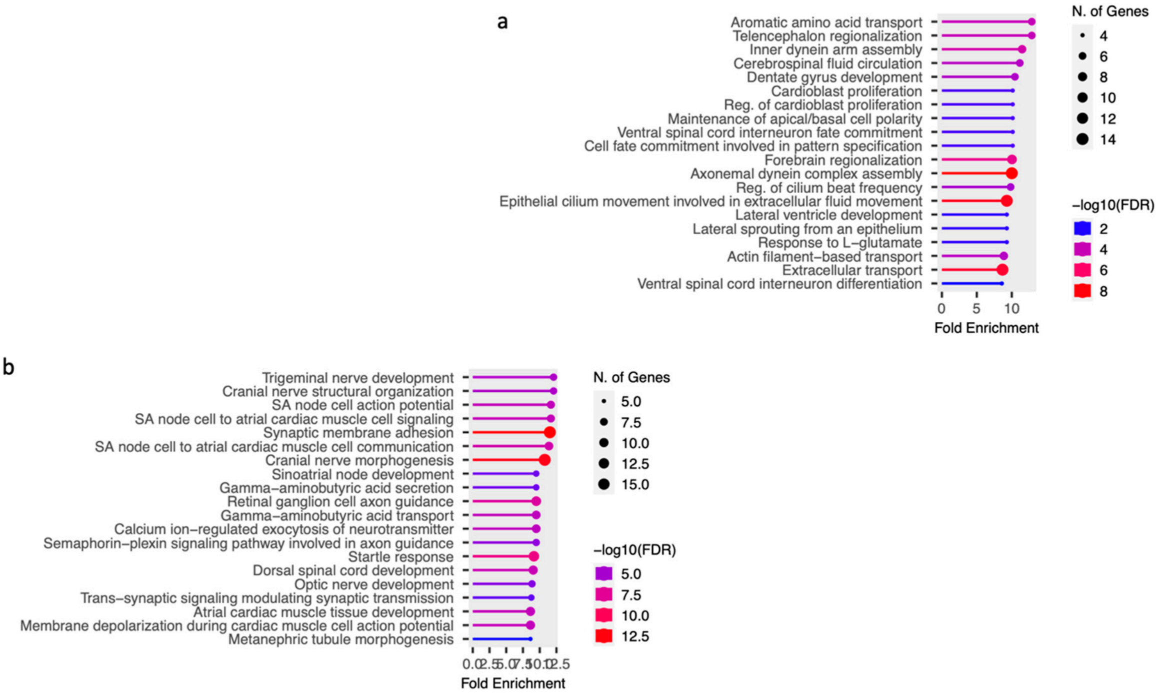

Figure S4. GSEA analysis of differentially expressed genes. (a) Differentially downregulated genes enrichment bar plot involved in different biological processes. (b) Differentially upregulated genes enrichment bar plot involved in different biological processes.

Table S5. Determination of differentially expressed secreted proteins after XIST silencing.

Table

https://aguedutr-my.sharepoint.com/:x:/g/personal/nihan_aktas_agu_edu_tr/EcSZAEyXRcdFuqLVMNBsruoB

I_82SBUoXy6VrPa_nUtQwg?e=sHX3lY&wdLOR=c7AD34110-3352-4378-953E-2B3CD8C831B2

Table S6. Gene set enrichment analysis of secreted proteins after XIST silencing.

Table

https://aguedutr-my.sharepoint.com/:x:/g/personal/nihan_aktas_agu_edu_tr/EcUUPJEzavNKk9tHP4P1IgUBIRYLXy0zPrXM6H6vGQv-wQ?e=DSKhah&wdLOR=c16313833-9DF3-4432-9EE6-FB90D818AC99
